# Competing pathways to aromaticity governed by amine dehydrogenation and metal–organic complexation in on-surface synthesis†

**DOI:** 10.1039/d4sc07550a

**Published:** 2025-01-13

**Authors:** Andrés Lombana, Songpol Chaunchaiyakul, Olivier Chuzel, Denis Hagebaum-Reignier, Jean-Luc Parrain, Franck Bocquet, Laurent Nony, Christian Loppacher, Federica Bondino, Elena Magnano, Hiroshi Imada, Emiko Kazuma, Yousoo Kim, Luca Giovanelli, Sylvain Clair

**Affiliations:** a Aix Marseille University, Université de Toulon, CNRS, IM2NP 13013 Marseille France luca.giovanelli@im2np.fr sylvain.clair@cnrs.fr; b Surface and Interface Science Laboratory, RIKEN 2-1 Hirosawa Wako Saitama 351-0198 Japan; c Aix Marseille Univ., CNRS, Centrale Med., ISM2 Marseille France; d CNR – Istituto Officina dei Materiali (IOM) AREA Science Park, Basovizza 34149 Trieste Italy; e Nanotechnology Research Laboratory, Faculty of Engineering, University of Sydney Camperdown 2006 Australia

## Abstract

We investigated the reactivity of a *gem*-dichlorovinyl-carbazole precursor in the on-surface synthesis approach. Our findings reveal that, on the Au(111) surface, the thermally-induced dehalogenation reaction led to the formation of cumulene dimers. Contrastingly, the more reactive Cu(111) surface promoted the formation of a polyheterocyclic compound exhibiting extended aromaticity. The latter was found to be related to the dehydrogenation of the amine groups, which did not occur on Au(111), thus promoting the different reactivity observed. At higher annealing temperature, selective C–H activation led to the formation of well-defined organometallic chains. In addition, we found that the amine complexation with metal adatom on Cu(111) was an inhibiting factor for the dimerization reaction, a challenge that could be overcome through proper control of the deposition conditions.

## Abstract 2

Two distinct reaction pathways are observed on metal surfaces to preserve the dimer aromaticity.

## Graphical abstract

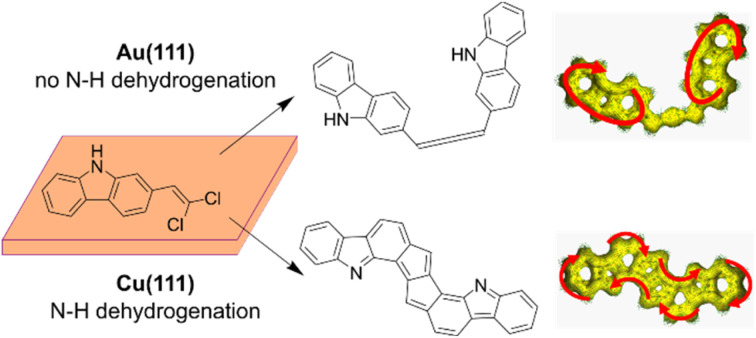

## Introduction

Well-defined oriented coinage metal surfaces in ultrahigh vacuum (UHV) are ideal platforms to study the fundamental chemical mechanisms in the on-surface synthesis approach.^1–5^ Here, the particular role of the substrate, acting as an atomically-defined template and steering molecular confinement in a two-dimensional environment, can give rise to unexpected behaviors which deviate from what is known from standard chemistry in the liquid phase.^6–9^ With the help of advanced experimental characterization tools, and in particular scanning probe microscopy, exquisite details can be unveiled on the diversity of behaviors of organic systems at the single molecule level.^10–12^ The reactivity in on-surface synthesis is particularly hard to predict because a large number of interface effects can arise.^2^ Furthermore, depending on the design of the precursor, competing reaction pathways can be selectively activated, for example in domino-type reactions.^13–18^

Aromaticity is a widely used concept in chemistry.^19–22^ It refers to enhanced stability, homogeneous structure and also sets a criterion for reactivity. The aromaticity of a molecule is steered by the number of π-electrons injected into its structure. In the case of nitrogen-containing compounds with sp^2^ hybridization, the aromaticity can be readily controlled depending on whether the nitrogen atom is of pyrrolic or pyridinic/azafulvene type. In the former case, the presence of an in-plane N–H bond drives the implication of the nitrogen atom lone pair into the π-electron system, while in the latter case the lone pair is of σ-character and only one π-electron is provided. Amino groups are well-known to get easily dehydrogenated upon adsorption on a metal surface,^23–27^ thus providing a direct way of tuning the provision of π-electrons and, in turn, the aromaticity of the molecular system.

On a surface, the aromaticity and other related effects have been recently proposed as important concepts contributing to the molecular reactivity. The regioselectivity in cyclization reactions was in this way explained by the aromatic pathway in free-base porphyrins^28^ and in azulene moieties.^29^ The hydrogenation state of nitrogen atoms was predicted to provide a way to control the electron doping in graphene nanoribbons and to induce important modifications in their electronic properties.^30^ The charge state in pentacene-dione and tetrone could be tuned by the nature of the supporting substrate,^31^ or in porphine directly by tip manipulation in scanning probe microscopy,^32^ providing a way to control the aromaticity and the related structural modifications in these compounds. Finally, the degree of charge transfer from the metal surface was shown to play a dominant role in the doping level of aza-triangulene, providing it with either a close- or an open-shell structure.^33^

**Fig. 1:**
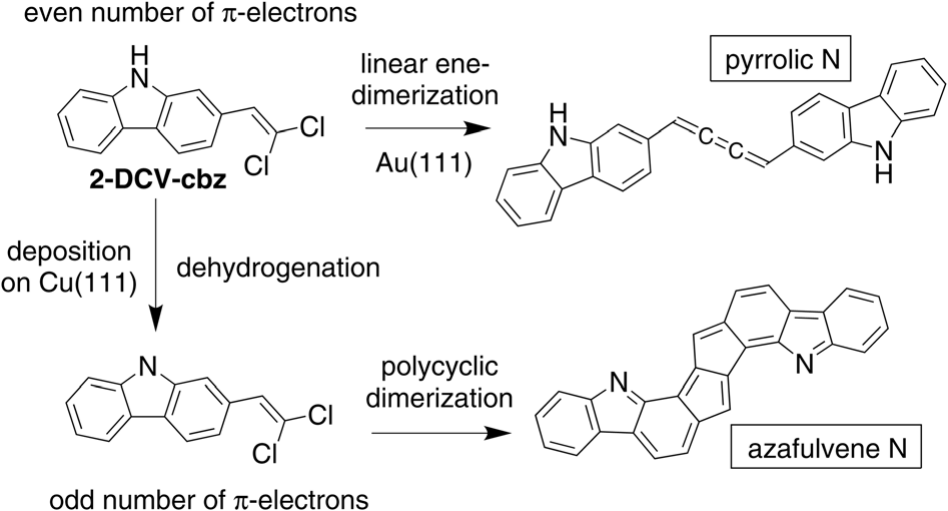
General scheme showing the complex reactivity of 2-DCV-cbz on the Au(111) and Cu(111) metal surfaces. Depending on the pyrrolic or azafulvene type of the N atom, the dimerization reaction is selective towards the preservation of the aromaticity in the product.

In this work we have investigated the link between the dehydrogenation state of nitrogen and the aromaticity in the on-surface reactivity. We have selected 2-(2,2-dichlorovinyl)-9*H*-carbazole (2-DCV-cbz, see Fig. 1) as a test molecule for several reasons. Carbazole derivatives are widely used in organic electronics^34–38^ due to their ease of structural modification, their high stability, and their aromaticity. On the other hand, their *gem*-dihalogenoalkene derivatives were shown to be highly reactive towards the copper-promoted formation of free base ynamines and ynamides, which are subgroups of alkynes directly substituted by a nitrogen atom.^39–41^ On the Au(111) surface, the reactivity of *gem*-dibromomethyl-containing molecules^42^ has been demonstrated in the form of a dehalogenative homocoupling reaction to create double^43^ or triple^44–47^ C–C bonds. Cumulene formation has been described from *gem*-dibromoalkenes^42^ on Au(111)^48,49^ and Cu(110).^50^ The 2-DCV-cbz precursor is also expected to interact with the intrinsic metal adatoms that are diffusing on the surface. In general, stable molecule–metal complexes are readily obtained on a surface for molecules containing electron-withdrawing groups, leading eventually to the formation of extended self-assembled networks.^51,52^ Moreover, organometallic compounds are usually found as intermediate sates in the Ullmann-like coupling reaction of halogenated species.^53^

The on-surface reactivity of the *gem*-dichlorovinyl-carbazole 2-DCV-cbz precursor is thus potentially rich in terms of competing pathways. In this work, we found that the acceptorless dehydrogenation reaction^54^ of the secondary amine can be used as a reference governing the global selectivity (see Fig. 1). More precisely, the homocoupling reaction initiated by the dehalogenation of the dichlorovinyl group is limited to the formation of a cumulene unit when the N–H bond is preserved. In contrast, on a more reactive surface, the dehydrogenation of the amine can occur and drive an unexpected domino reaction leading to the formation of a polyheterocyclic compound with extended aromaticity. The different reactivities of the metal surface toward the amine dehydrogenation was provided by the Au(111) or the Cu(111) surfaces. We also observed that on Cu(111) the complexation of the nitrogen atom with metal adatoms is an inhibiting factor for the dimerization reaction, which can be overcome through proper control of the deposition conditions. The different surfaces were characterized at the single molecule level by scanning tunneling microscopy (STM), in combination with synchrotron-radiation core level photoemission spectroscopy (XPS). Theoretical calculations at the density functional theory (DFT) level were performed to support the experimental data, and anisotropy of the current induced density (ACID) calculations to characterize the aromaticity of the reaction products.

## Results and discussion

### Precursor synthesis

**Fig. 2:**
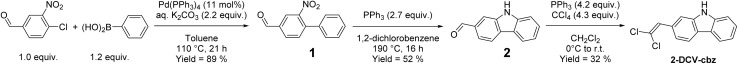
Three-step synthesis of the precursor 2-DCV-cbz.

2-(2,2-Dichlorovinyl)-9*H*-carbazole (2-DCV-cbz) was achieved in a three-step synthesis (Fig. 2, Experimental details in ESI†). In a classical approach to carbazoles, a Suzuki–Miyaura cross-coupling was performed to obtain the 2-nitro-(1,1′-biphenyl)-4-carbaldehyde 1, followed by a phosphine-mediated reductive cyclization yielding to the 9*H*-carbazole-2-carbaldehyde 2, accordingly to the literature.^55^2-DCV-cbz was then obtained through a classical Ramirez–Corey–Fuchs reaction in moderate yield.^39^

### On-surface synthesis

**Fig. 3:**
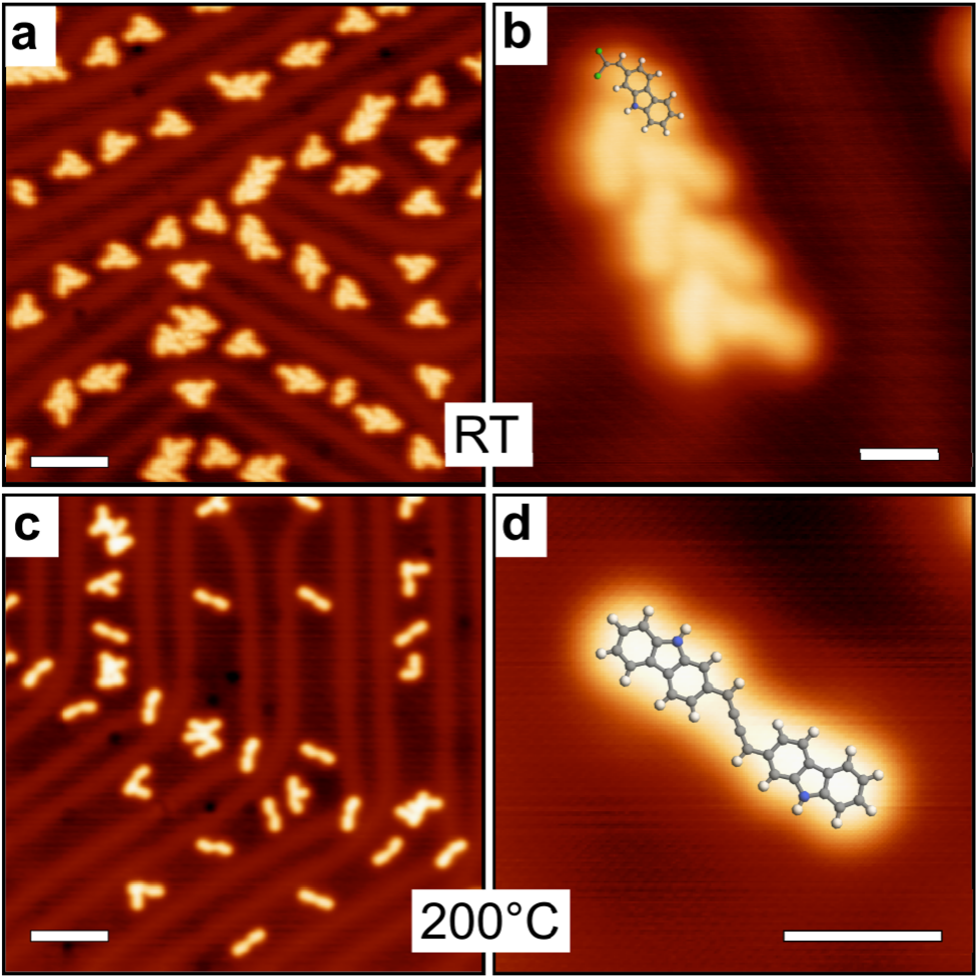
LT-STM images of 2-DCV-cbz obtained on Au(111). (a and b) Directly after deposition at room temperature the molecules are preserved and organized in small clusters in chevron fashion. (c and d) After annealing at 200 °C the homocoupling reaction is activated, leading to the formation of cumulene dimers. Scale bars: (a and c): 5 nm, (b and d): 1 nm.

As observed by low temperature STM (LT-STM), the room-temperature deposition of 2-DCV-cbz on the low reactive Au(111) surface at low coverage results in the formation of small aggregates preferentially adsorbed on the fcc regions of the reconstructed surface (Fig. 3a and b). These aggregates are composed of small rods corresponding to individual molecules self-assembled in chevron fashion. XPS data confirm that the integrity of the precursors is preserved (see below). The aggregates are probably stabilized through halogen and Cl–H bonding.^56,57^

After annealing at 200 °C, different aggregates are observed by STM. Their shape and size correspond to the formation of cumulene dimers (Fig. 3c and d). The different configurations could be well identified from the STM images (see ESI Fig. S1a–c†). This reaction has been reported at room temperature with similar alkenyl *gem*-dibromide precursors,^48,58^ highlighting the high reactivity of these groups towards the formation of cumulene units. XPS data further confirms the reaction product and the C–Cl bond scission. Beside the cumulene dimers, other more complex oligomeric structures are found that can be attributed to the additional formation of ynamine links (see ESI Fig. S1d and e†). These compounds form in a small minority, as confirmed by XPS data showing that the chemical structure of the carbazole macrocycle and the secondary amine N–H group remain largely unaffected.

**Fig. 4:**
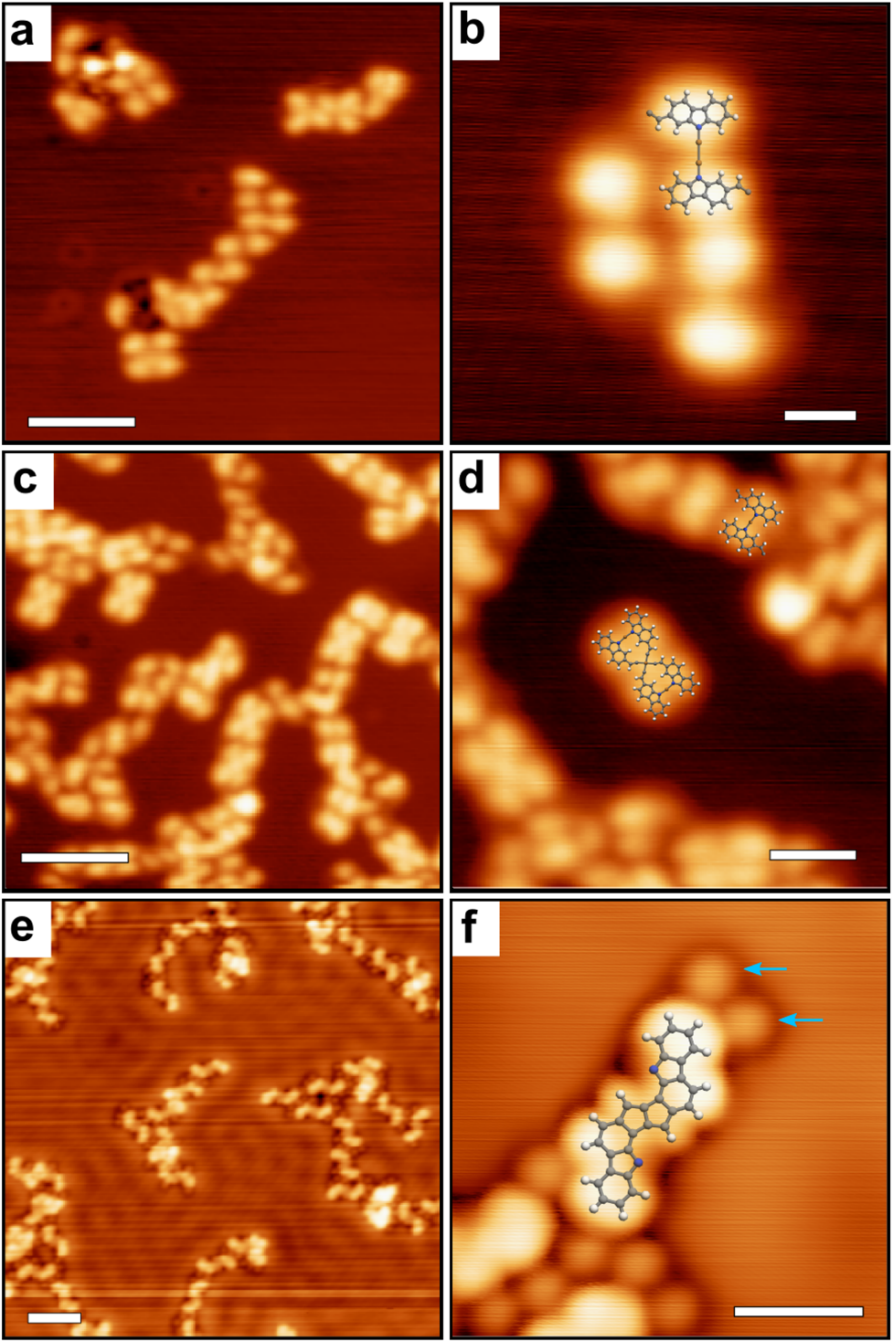
LT-STM images of 2-DCV-cbz on Cu(111). (a and b) RT deposition. (c and d) Annealed to 200 °C. (e and f) Hot substrate (200 °C) deposition. In (f) the chlorine atoms (blue arrows) are visible in the vicinity of the dimer. Scale bars: (a, c and e) 5 nm, (b and f) 1 nm, (d) 2 nm.

On the more reactive Cu(111) surface the situation changes drastically. Fig. 4a and b shows LT-STM images of the surface obtained directly after deposition at room temperature. Small clusters are observed, with a tendency to form linear assemblies. As highlighted in Fig. 4b (see also Fig. S2†), the basic bonding unit is a dimeric compound probably corresponding to a bis(*N*,*N*)-carbazole-copper complex derivative, similar to what was previously observed on Ag(111).^59^ XPS data identifies the dehydrogenation of the N atomic sites and their possible complexation with Cu adatoms (see below). The distance between two metal-coordinated N atoms in the superimposed model of Fig. 4b is about 7 Å, which suggests that the number of bridging copper adatoms in the complex can be multiple.^60–64^ At room temperature these species are highly mobile, suggesting a relatively low interaction with the metal surface (see ESI, Fig. S3†). The chlorine atoms are detached from the molecule, as shown by XPS below, and the vinylidene carbene^65^ is probably stabilized by the Cu surface.^48,50,66^ The vinylidene carbene can possibly transform into an alkyne through a Fritsch–Buttenberg–Wiechell rearrangement,^67^ as it has been proposed for another similar derivative.^68^ Both structures are undistinguishable in the STM images (see ESI Fig. S4†).

Surprisingly, the detached Cl atoms are not directly visible at this stage in the STM images. This observation may be due to a particular tip state and imaging conditions, but it can be also intrinsically related to the configuration of chlorine in interaction with the molecular structure. Indeed, in previous work relating the on-surface formation of alkyne^44,47^ or stilbene links,^43^ at least part of the released halogens seemed to be missing or similarly transparent in the STM images.

Importantly, the bis(*N*,*N*)-carbazole-copper complex derivatives are stable up to an annealing temperature of 200 °C (Fig. 4c and d). After annealing, the distance between two metal-coordinated N atoms in the superimposed model is about 4 Å, which suggests that one single copper adatoms is involved in the complex. In addition to the dimers, tetrameric structures are observed (Fig. 4d). Here also, the STM data cannot conclude on a possible Fritsch–Buttenberg–Wiechell rearrangement,^67^ because both vinylene-based and alkyne-based tetramers are indistinguishable in the images (see ESI Fig. S5†).

Alternatively, the 2-DCV-cbz precursors were deposited directly on the hot Cu(111) surface kept at 200 °C. This leads to a completely different behavior as shown in Fig. 4e and f. Here the basic unit can be ascribed to a covalent dimer, where the carbazole precursors are linked through a fused pentalene unit, leading to a pentaleno[1,2-*a*:4,5-*a*′]dicarbazole. The formation of fused pentalenes is not unusual in on-surface synthesis when polyradicals are available, as observed on Cu(111),^9,69^ Ag(110)^70^ and Au(111).^8,71–74^ Here, the dimeric adduct is probably obtained through a domino Fritsch–Buttenberg–Wiechell rearrangement^67^ followed by a dimerization reaction, both assisted by copper adatoms. The individual dimers tend to be positioned in the vicinity of each other in linear fashion, probably through weak van der Waals interaction. The nitrogen atoms are most probably not coordinated with a copper adatom, as it will be discussed in the text below. Close to the dimers, the released Cl atoms are now clearly visible. They appear well-separated from the molecules, in weak interaction as they remain in their vicinity. At room temperature all structures are still highly mobile on the surface (see ESI, Fig. S3†).

**Fig. 5:**
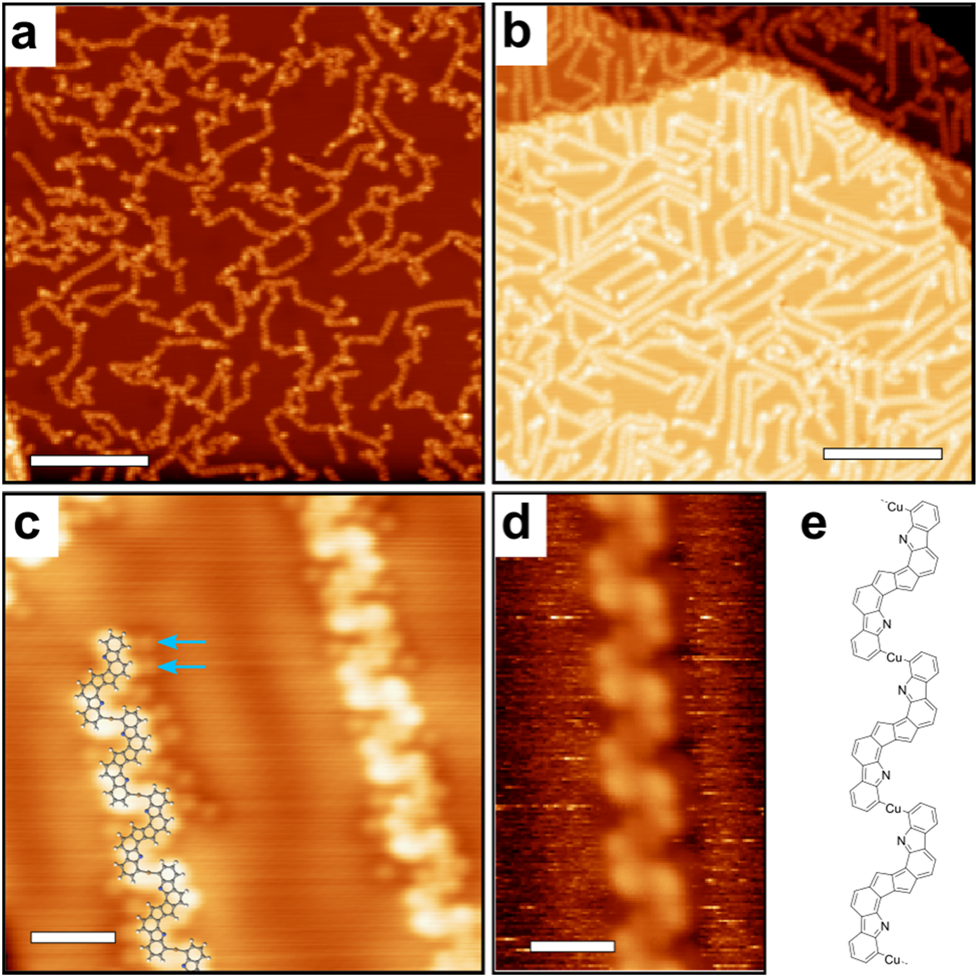
STM images of 2-DCV-cbz on Cu(111) annealed to 300 °C following deposition (a) at room temperature or (b–d) at 200 °C. (e) Schematic structure of the polymeric chains. The images were acquired at low-temperature (a–c) or at room temperature (d). In (c) the chlorine atoms (blue arrows) are visible in the vicinity of the chains. Scale bars: (a and b) 20 nm, (c and d) 1 nm.

Finally, Fig. 5 shows STM images obtained after annealing to 300 °C. Two different configurations are found. In the case of room temperature deposition followed by annealing, linear structures are observed but of limited size and highly disordered (Fig. 5a). In the case of direct deposition on a hot substrate (200 °C) followed by further annealing at 300 °C, well-defined 20 to 30 nm long polymeric chains are observed (Fig. 5b). Inside the chains, the repeating units consist of fused-pentalene dimers (as also observed in Fig. 4f) that are further connected through the formation of an organometallic complex (C–Cu–C) due to an additional C–H bond activation step in *ortho* position. Similar C–H bond activation leading to an organometallic coupling has been previously observed.^24,25,75–77^ In principle, the coordination could also be made through the formation of a double C–Cu–N bridge (see ESI Fig. S6†). This would however produce a polymer with shorter periodicity as compared to the experimental one (1.35 ± 0.1) nm and can thus be excluded. Note that such C–Cu–N bridge motif could be observed sporadically right after deposition at 200 °C (see ESI Fig. S7†) but is not stable at 300 °C in the final polymer structure. The proposed structure is further confirmed by XPS (see text below). In the vicinity of the chains the released Cl atoms are still visible (Fig. 5c). At room temperature the chains are stable (Fig. 5d and ESI Fig. S3†), only the chlorine atoms are not imaged anymore due to fast diffusion.

The chains present scarcely threefold interconnections with the local formation of trimeric compounds (see ESI, Fig. S8†). Due to the manifold configurations observed and to the high flexibility of the structures, it was not possible to elucidate the precise chemical structure of these branching motifs from the STM images. Other kinds of defects are observed, in the form of point defects, or locally curved sections (see ESI, Fig. S8†), which could similarly not be precisely characterized.

A temperature of 200 °C is sufficient to activate the covalent cyclization reaction to form the pentalene dimers, but the latter form exclusively when the precursors are deposited directly on a hot substrate. When the surface is annealed from RT to 200 °C, only the bis(*N*,*N*)-carbazole-copper complexes obtained already at room temperature are present. As a matter of fact, it is not possible to form flat pentalene dimers from the metal–organic N–Cu–N complexes due to the steric hindrance between the neighboring hydrogens^78^ (ESI Fig. S9†). Once the metal–organic complex is formed, the covalent dimerization reaction is thus activated at higher temperature (between 200 °C and 300 °C) and in this case the limiting step is the dissociation of the complexes. The formation of the metal–organic complexes represents then an important kinetic blockade in the global reaction pathway, leading to a low quality of the final polymeric product with short and defective chains (Fig. 5a). Alternatively, the blockade can be overcome by depositing the precursors directly at 200 °C thus inhibiting the formation of the metal–organic complexes. This pathway leads to a high quality of the final polymeric product with long and straight chains (Fig. 5b).

### XPS data

**Fig. 6:**
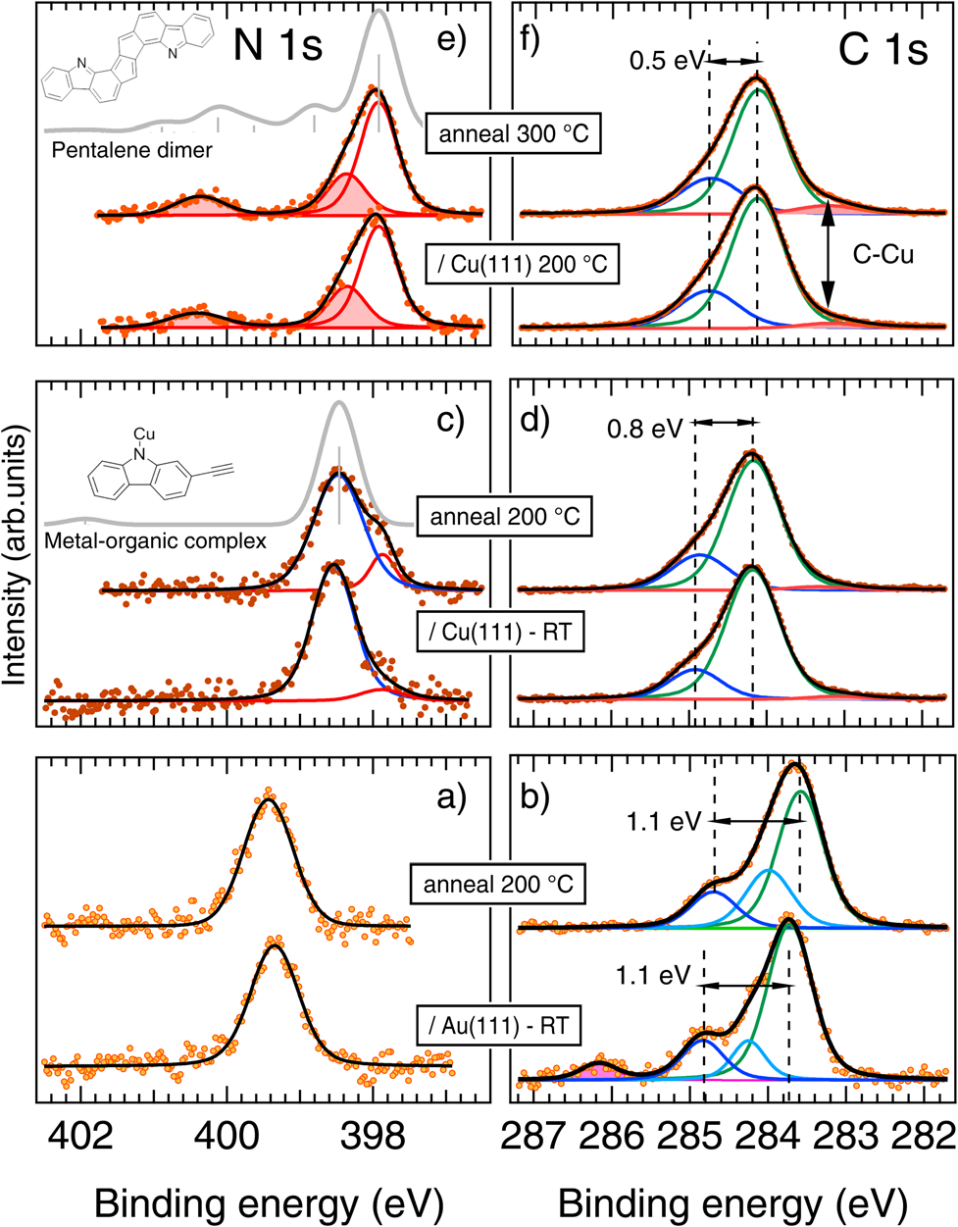
XPS results. (a, c and e) N 1s and (b, d and f) C 1s spectra of 2-DCV-cbz on Au(111) and Cu(111). (a and b) Deposition on Au(111) at RT followed by annealing at 200 °C. (c–f) For Cu(111) two sets of spectra are displayed according to substrate temperature during deposition. Panels (c) and (e) also display as gray lines the calculated spectra including the shake-up satellites for the metal–organic complex and the pentalene dimer.

To validate and refine the structural model of different phases, least-square fitting of XPS spectra was used, combined with DFT core level calculations (see Fig. 6 and ESI Fig. S12 and Table S1†).

#### Au(111) – pristine precursors and cumulene dimers

XPS data confirm that when adsorbed on Au(111) at RT, the precursor molecules remain in their pristine form. The N 1s (Fig. 6a), consisting of a single symmetric peak at 399.4 eV, is representative of a protonated pyrrole moiety.^79^ The C 1s (Fig. 6b) is composed by at least three contributions from chemically nonequivalent atoms. With the help of DFT (see ESI, Fig. S12†), the assignment can be performed by considering the chemical shift induced by the Cl and N heteroatoms. The highest binding energy (BE) component is due to the Cl–C–Cl atoms (286.2 eV, pink peak in the fit). The electron withdrawing effect of the chlorine atoms extend to the next two C atoms which contribute to the asymmetry of the main line (cyan component at 284.2 eV). On the other hand, the C–C–N atoms contribute to the shoulder to the main peak (284.8 eV, blue component). The main peak, centered at 283.7 eV, comes from the reminder of the two benzene rings.^80^ Finally, the Cl 2p spectrum (ESI Fig. S10†), typical of the Cl–C bond, confirms a pristine form for 2-DCV-cbz adsorption on Au(111).^81^ When the system is annealed at 200 °C the dimerization through cumulene formation is observed in STM. The main difference in XPS spectra is the disappearance of the Cl–C–Cl component in C 1s, accompanied by the disappearance of the Cl 2p signal and implying a detachment of the Cl atoms from the 2-DCV-cbz molecules and subsequent desorption. On the other hand, the N 1s spectrum is essentially unmodified indicating the persistence of the N–H bonding. Apart from the loss of the Cl–C–Cl component, the cumulene formation results in only minor changes in the C 1s spectrum, concerning essentially the Cl–C–Cl atom now being part of the cumulene bridge (C=C=C) and leading to an increase of the cyan component^49^ (see also ESI Fig. S12†).

#### Cu(111) – metal–organic complexes

When compared to Au(111), deposition at RT on Cu(111) shows a striking low BE core level shift (CLS) of the N 1s which is consistent with the substrate-induced dehydrogenation of an amino group^77,82,83^ and consequent promotion of N–Cu–N coordination, resulting in the metal–organic complexes observed by STM (Fig. 4a and b). Upon annealing at 200 °C, a minority component grows at low binding energy, foreshadowing the structural modifications observed at higher temperature (see below).

On the other hand, C 1s shows a higher BE for adsorption on Cu(111) with respect to Au(111). This can have different origins, mainly related to different molecule–substrate interactions.^84^ Although the interface interaction can have important consequences on the intermolecular ones, the promotion of intermolecular bonds and dimerization is better captured by the line shape analysis rather than by the BE rigid shift. In going from Au to Cu, the line shape of the C 1s changes considerably. The spectra are broader, meaningful of a larger number of non-equivalent C sites, but the spectra can be fitted essentially with two components. The changes with respect to adsorption on Au(111) can be summarized as being due to the disappearance of the vinyl dichloride component and to the reduced energy shift of the C–N component due to the electron donating effect of the Cu adatoms on the carbazole N and, in turn, on the C–N bond.^85^ Both these effects are captured by DFT calculation modeling (see ESI Fig. S12†). Finally, a minority component is added at low BE to improve the fit, probably due to defects.^80^ When the RT deposited molecules are annealed at 200 °C the C 1s spectrum is essentially unaffected.

The Cl 2p spectrum (Fig. S10†) shows a strong low BE shift with respect to the molecules deposited on Au(111), in agreement with reported data from atomic Cl/Cu(111),^86^ thus supporting the dechlorination at RT. Only minor intensity variations are observed upon annealing, indicating a strong Cl–Cu bond.

#### Cu(111) – polymeric chains

From the STM analysis, a different chemical modification of the precursors is expected when deposition is performed with the substrate kept at 200 °C. In fact, when analyzing the N 1s spectrum, the small low-BE component at 397.8 eV observed for RT deposition (Fig. 6c) becomes the dominant feature and two new features appear at higher BE (shaded-area peaks in the fit of Fig. 6e). In particular, the isolated feature at 2.4 eV higher BE from the main line reveals a fundamental aspect of the molecules forming the polymeric chains observed in STM. Such a feature is unlike to be due to a chemical shift since the only possible configuration would concern a re-hydrogenation and would result in a less shifted component.^77,79^ The new feature can then be assigned to a satellite of the main peak.

In XPS spectra, satellites to a main line are often observed as a consequence of final-state screening of the core-hole.^87^ In a thick molecular film (or in the gas phase), satellites (also named shake-up) originate essentially from monopole-allowed electronic transitions between the occupied and the unoccupied molecular orbitals. Generally, the excitations between frontier orbitals are favored with respect to higher energy transitions and their intensities depend on the energy gap as well as on the perturbing effect of the core-hole on the orbital involved.^87–89^ The situation is somehow different when the molecules are adsorbed on a surface because the interface can participate to the core-hole screening.^90^ Since the molecule–substrate interaction is sometimes located at specific atoms within the adsorbed molecule, the final state effects can vary according to the particular core level being excited.^91^ Moreover, for a given core level, asymmetries due to the presence of metallic-hybrid interface-states can be observed together with shake-up features in chemically shifted components.^92^ Nevertheless, for weak interface interaction, the adsorbate may display shake-up features very close to that observed in the gas phase or thick film.^93,94^

To reproduce the occurrence of the shake-up features in our system we have modeled the final state effects in an equivalent core-hole (ECH) TD-DFT approximation^95^ for gas-phase molecules, assuming that the substrate plays a negligible role. The pristine precursor has a rather large HOMO–LUMO gap in the ground state, and the related satellite feature is vanishing, as shown in ESI Fig. S13.† The metal–organic complexes observed at RT have a reduced HOMO–LUMO gap, but the calculations confirm that no satellite is expected (gray line in Fig. 6c). Conversely, when the pentalene dimer is considered, several satellites appear in the model spectrum (gray line in Fig. 6e). This suggests a strong perturbation of the electronic structure when creating a core hole on the N atom and the promotion of sizeable interband transitions.^87^ Most importantly, no satellite structure would appear in the model spectrum of the hydrogenated dimer (ESI Fig. S14†) due to the reduced delocalization (loss of aromaticity) and consequent increased energy gap (see the ACID calculation results below).

The presence of shake-up satellites can also explain the low BE shift of the main line: the presence of intense shake-up features is concomitant with an efficiently-screened core-hole^89,96^ (essentially through intramolecular excitations) and thus with a low BE shift of the main line. When the N 1s position is calculated by the first momentum sum rule,^96^ the virtual unscreened core level is found at 398.3 eV, implying a minor BE shift with respect to the metal–organic complexes obtained at RT (centered at 398.45 eV). The molecule–substrate interaction is then not sensibly different for the two systems.

Additional insights on the structural details of the polymeric chains can be inferred by the C 1s spectrum. Upon deposition at 200 °C (Fig. 6f), the small changes already observed after annealing the RT-deposition sample are enhanced, namely: (i) the C–N component gets closer to the C–C one and (ii) a new feature at low BE (vertical arrow) appears which can be ascribed to the C–Cu sites. Both these features can be reproduced by DFT calculations of the polymeric chains as compared to the metal–organic complexes (see ESI Fig. S12†) and confirm the structure proposed for the final polymer formed at high annealing temperature (Fig. 5e).

The two sets of spectra obtained after annealing at 300 °C by the two different deposition methods, RT deposition or hot substrate deposition, are identical (see ESI Fig. S11†). This indicates that the two different preparation methods provide the same chemical state of the products after 300 °C annealing. Only structural differences are found in the STM images, with a much higher quality of the chains in the case of hot substrate deposition. The absence of clear satellite features in the C 1s spectra, as compared to N 1s, can be due either to a different final-state screening or to the concomitant presence of several chemically-shifted shake-up structures.

### Assessment of the aromaticity

**Fig. 7:**
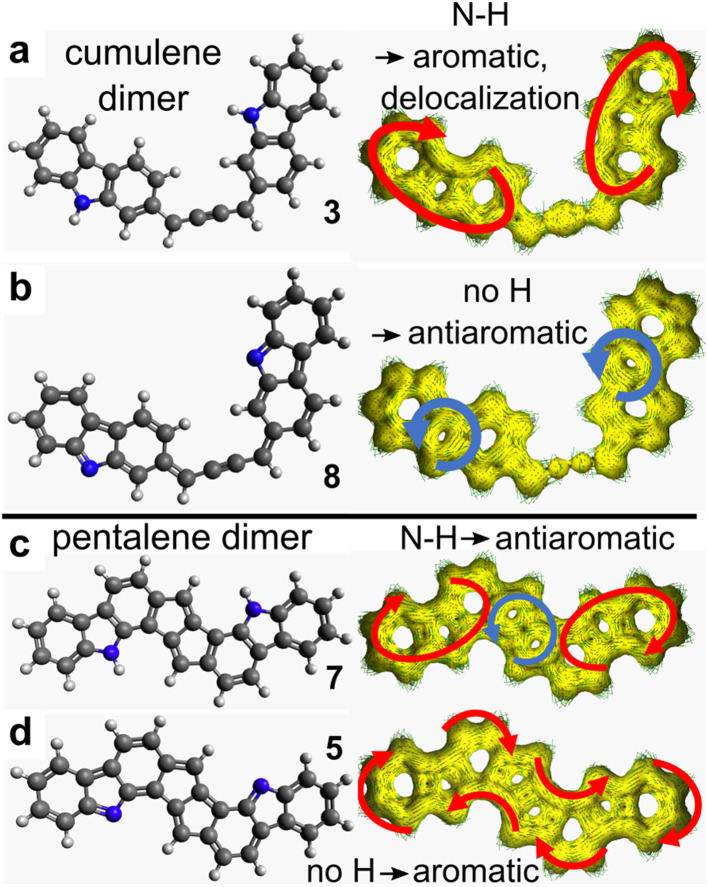
ACID plots (right) computed for different dimer structures. The red (blue) arrows indicate the clockwise (anti-clockwise) circulation direction of the π-electrons, representative of the aromatic (antiaromatic) character, respectively. (a) The hydrogenated cumulene dimer (3) exhibits an aromatic character with full delocalization. (b) The dehydrogenated cumulene dimer (8) exhibits an antiaromatic pattern on the carbazole units. (c) The hydrogenated pentalene dimer (7) is aromatic on the carbazole cores but antiaromatic on the pentalene. (d) The dehydrogenated pentalene dimer (5) shows global aromaticity over the whole structure.

ACID calculations^97^ were performed for different dimeric structures to assess their aromatic character (Fig. 7). The cumulene (3) and pentalene (5) dimers, as observed on the surface, show a pronounced aromatic character represented by a clockwise circulation of the π electrons. In particular, for the dehydrogenated pentalene dimer (5) the π electron circulation ring is positioned over the entire molecular skeleton. In contrary, for the virtual dimers (7) and (8) presenting an opposite hydrogenation state of the nitrogen site, an antiaromatic character appears in the form of anti-clockwise circulation rings.

Because the aromaticity of (5) is represented by a unique macrocycle delocalized over the whole dimer (Fig. 7d), it is legitimate to assess Hückel's rule in this polycyclic compound. There are 14 C and 1 N atoms in the precursor molecule. For the pentalene dimer (5), the lone pair of N is localized in-plane and N thus provides only 1 π-electron. Accordingly, the dimer bears (14 C + 1 N) × 2 = 30 π-electrons, *i.e.* 4*n* + 2 with *n* = 7. In case the nitrogen atom is hydrogenated as in the virtual dimer (7), the lone pair of the nitrogen atom would provide 2 π-electrons, so in total (14 C + 2 N) × 2 = 32 = 4*n* π-electrons with *n* = 8. Hückel's rule therefore correctly predicts that the pentalene dimer is aromatic (antiaromatic) in the case of N being dehydrogenated (hydrogenated), respectively. For the hydrogenated compound (7), the ACID plot shows partial delocalization on each carbazole unit and on the pentalene core (Fig. 7c). Here it is certainly less justified to consider the whole skeleton with its 30 electrons. Rather, one should count 14 = 4 × 3 + 2 (4*n* + 2) electrons on each carbazole (aromatic) and 8 = 4 × 2 (4*n*) electrons on the pentalene core (antiaromatic). In this virtual compound, the aromaticity of the hydrogenated carbazole is therefore preserved and the antiaromatic character is limited to the pentalene unit.^74^

## Discussion

**Fig. 8:**
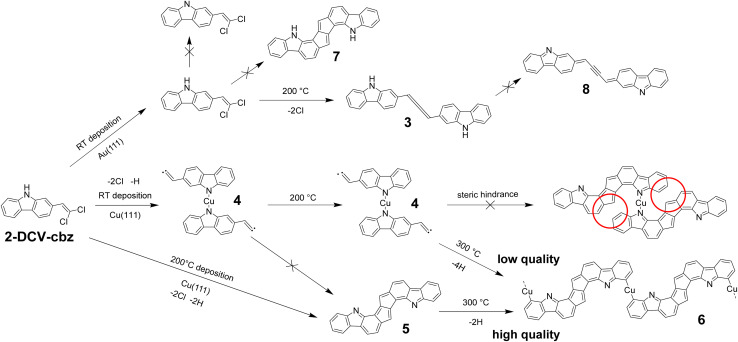
General reaction scheme of 2-DCV-cbz on the Au(111) and Cu(111) surfaces. The vinylidene carbene in (4) is possibly rearranged into an alkyne.

The global behavior of 2-DCV-cbz on Au(111) and Cu(111) is summarized in Fig. 8. On Au(111) at room temperature the precursors remain intact on the surface. In particular no change in the oxidation state of the N site is found. After annealing, dehalogenation occurs leading to the formation of individual cumulene dimers (3). In the latter the N–H bond is still preserved thanks to the low reactivity of Au(111) and also due to the fact that the dehydrogenation reaction leading to (8) is not favorable, because it would provide an antiaromatic character to the carbazole units, as shown by ACID calculations (see Fig. 7b).

On Cu(111) the high reactivity of copper leads to the formation of a vinylidene carbene and to the dehydrogenation of the amine moiety. Two distinct scenarios are taking place depending on the reaction conditions. At room temperature the dehydrogenated carbazoles form the dimeric metal–organic complexes (4) through coordination with Cu adatoms. These compounds are stable up to a temperature of 200 °C and completely inhibit the reactivity of the vinylidene carbene moiety. The homocoupling reaction can be activated in two ways, either by further annealing to a temperature of 300 °C, or by a direct deposition onto the surface kept at 200 °C, thus avoiding the prior formation of the metal–organic complexes. As mentioned earlier, the covalent dimerization leading to the pentalene dimer (5) probably occurs through a domino Fritsch–Buttenberg–Wiechell rearrangement followed by a dimerization reaction, both assisted by copper adatoms. At 300 °C we observe furthermore the activation of the C–H bond in *ortho*-position of the N site and the extensive formation of the linear coordinative organometallic polymer (6).

The pentalene dimer (5) exhibits a pronounced aromatic character fully delocalized over the whole compound and is thus highly stabilized. This is shown by the presence of a strong shake-up satellite at the N 1s core level (Fig. 5e) and by the ACID calculations (Fig. 7d). Remarkably, if the nitrogen atoms were hydrogenated as in (7), the aromaticity would be restrained to the carbazole cores and the pentalene unit would be antiaromatic (Fig. 7c). Moreover, no shake-up satellite would be observed by XPS in that case (see ESI, Fig. S14†). Such situation would be highly unfavorable, which can explain the difference in reactivity observed between Au(111) and Cu(111). On Au(111) the pentalene formation is inhibited by the preservation of the N–H bond and the formation of a cumulene dimer is favored. On Cu(111), thanks to the dehydrogenation of the nitrogen atom, the aromatic pentalene dimer (5) is very stable and the formation of a cumulene, virtually antiaromatic (Fig. 7b), is unlikely.

The STM images of the pentalene dimers (Fig. 4e, f and 5), as well as the BE of the corresponding N 1s spectrum (Fig. 6e)^79^ cannot conclude on the possible complexation of the N site with Cu adatoms. However, the simulations of the shake-up feature predict that it should be quenched or at least importantly reduced in the case of complexation (see ESI Fig. S14†). We can thus postulate that the final polymer chains are not coordinated at the N sites, only at the C sites.

Finally, we can propose a scenario to explain the kinetic blockade observed on Cu(111) for the RT deposition. In the metal–organic complex (4), due to the coordination to Cu adatom the N lone pair is out-of-plane and engaged in the π-electron system. This impedes the formation of the pentalene dimer (5), for which the N lone pair is in-plane and not included into the π-electron system. Furthermore, the covalent dimerization is simply not sterically allowed in the planar configuration of the metal–organic complex^78^ (see red circles of Fig. 8 and ESI Fig. S9†). The energy barrier to achieve covalent dimerization from the metal–organic complex is probably overcome in the same temperature range as the C–H activation in *ortho*-position of the N site that is required in the formation of the final polymeric chains. This explains why the quality of the 1D chains is lowered as compared to the hot substrate deposition conditions. In the latter case, the C–H activation can occur after full completion of the dimeric structure, thus enhancing its selectivity and the quality of the polymeric chains.

## Conclusions

We investigated the reactivity of a *gem*-dichlorovinyl-carbazole precursor on the Au(111) and Cu(111) surfaces. The acceptorless dehydrogenation reaction of the amine moiety was shown to be the decisive parameter governing the selectivity of the homocoupling reaction. The cumulene dimer (3) is formed on the Au(111) surface, which preserves the N–H bond, while the pentalene dimer (5) is formed on the more reactive Cu(111) surface, which induces N–H dehydrogenation. The aromaticity of the respective dimer products is proposed as the driving force leading to the selection of the reaction pathway. This effect is explained in terms of number of π-electrons injected, depending whether the N lone pair is in-plane (azafulvene-type N) or out-of-plane (pyrrolic-type N), as defined by the hydrogenation state of the carbazole precursor. The presence of the shake-up features in the N 1s XPS spectrum was used as a signature of an extended aromaticity in the pentalene dimer. In addition, we found that the complexation of carbazole with Cu adatom is a kinetically limiting factor, strongly reducing the quality of the polymeric chains formed by high temperature annealing. This kinetic limitation can be overcome by the use of adequate growth conditions (hot substrate deposition).

In conclusion, we have shown that the dehydrogenation state of carbazole is a decisive factor governing its reactivity toward the homocoupling reaction, as can be inferred by considering the aromaticity of the different products. The present results confirm the key role played by the aromaticity in directing the on-surface reaction. Together with other recent works,^28–33^ they show that the population of π-electrons in polycyclic aromatic hydrocarbons (PAH) represents a seminal issue governing their aromaticity and in turn their stability, their electronic properties and their reactivity. We can expect that the issue of aromaticity will gain a growingly important place in the chemistry of surface-supported systems, thus extending further the amazing synthetic toolbox of on-surface synthesis.^2^

## Methods

### Chemical synthesis

All details in ESI.†

### STM

The experiments were performed in two different experiment setups in ultrahigh vacuum environment (UHV), with a base pressure in the low 10^−10^ mbar range. The single-crystal Cu(111) and Au(111) surfaces were cleaned by several cycles of Ar^+^ bombardment followed by annealing. The precursor was thermally sublimated from an evaporator heated to 75 °C for typical dosing time of 15 min providing a coverage of ∼0.5 monolayer. The STM images were acquired in constant current mode with typical tunneling current *I*_T_ ≈ 0.3 nA and sample bias *V*_T_ ≈ −(1 − 1.5) V. The room temperature STM measurements (RT-STM) were performed with a commercial Omicron VT-STM system. The low temperature measurements (LT-STM) were performed with a commercial Omicron LT-STM system operating at 4.7 K. All images were subsequently calibrated using atomically resolved images of the pristine surfaces. Images were partly treated with the free software WSxM^98^ and LMAPper.^99^

### XPS

Photoemission measurements were performed with a Scienta R3000 analyzer at normal emission and linearly horizontal polarized synchrotron radiation at the CNR-IOM BACH beamline at Elettra, Italy. The photon energy used for C 1s, Cu 3p and Cl 2p was 380 eV, and 510 eV for N 1s with a 0.2 eV total energy resolution. The base pressure of the measurement chamber was 7 × 10^−10^ mbar. The fit of the spectra was performed using KolXPD software with Voigt line shapes and a Shirley background. The fitting parameters are reported in the ESI.†

### Theoretical modeling

Theoretical calculations were carried out at the gas phase using Gaussian 16 software package.^100^ The polymer and the tetramer structures were optimized with the semi-empirical method PM6 (Parameterized Model number 6).^101^ For the evaluation of the core level shifts, the different structures were optimized using density functional theory (DFT) method with B3LYP hybrid functional^102^ and 6-311++G(2d,p) basis set, and the energy levels were evaluated with a 6-311 G(2d,p) basis set using natural bond orbitals.^103^ For the evaluation of the satellite features, the CAM-B3LYP functional^104^ and a 6-311G(2d,p) basis set was used.

The shake-up satellite bands corresponding to the excitation of the nitrogen 1s core orbitals were obtained following a two-step procedure. First, the valence electron excitations were calculated using the time-dependent density functional theory (TD-DFT), while the core-hole is described by the equivalent core approximation (ECH), also called *Z* + 1 approximation.^95,105^ This consists in replacing the core-ionized nitrogen atom by an oxygen atom with a positive charge and computing the ground electronic state of the corresponding ion. The excitation energy of a shake-up state is then related to the optical excitation energy of the core-ionized system. Second, the band intensities, proportional to the overlap between the ground and final states, were obtained following Martin and Shirley.^106^

The geometry of the pentalene and the cumulene dimers of Fig. 7 were optimized using DFT with B3LYP hybrid functional^102^ and 6-311++G(2d,p) basis set. From these geometries, ACID calculations (Anisotropy of the Current Induced Density) were performed using the B3LYP/Def2-TZVP level of calculation.^97^ The continuous set of gauge transformation (CSGT) method was used to calculate current densities,^107^ as implemented in the Gaussian16 package.^100^ The calculated current density was subsequently transformed in a rectangular grid and visualized as a cube-file with POVRay.^108^ The ACID method allows the visualization of an isosurface on which the induced current density vectors are plotted, showing diatropic (clockwise, aromatic) and paratropic (counterclockwise, antiaromatic) currents. The applied magnetic field is orthogonal to the molecular plane, pointing towards the observer in all illustrations. All ACID plots were generated using an isovalue of 0.03.

## Data availability

Experimental details can be found in the ESI.† All other data will be available on request.

## Author contributions

Conceptualization: O. C., J. L. P., L. N., C. L., Y. K., S. C.; funding acquisition: O. C., J. L. P., L. N., C. L., Y. K., L. G., S. C.; investigation: A. L., S. C., L. N., C. L., F. B., E. M., H. I., E. K., L. G., S. C.; formal analysis: A. L., S. C., O. C., D. H. R., F. B., L. G., S. C.; writing – original draft: L. G., S. C.; writing – review & editing: O. C., L. G., S. C. with contributions from all authors.

## Conflicts of interest

There are no conflicts to declare.

## Supplementary Material

SC-016-D4SC07550A-s001
