# Supplementary material for: Competing pathways to aromaticity governed by amine dehydrogenation and metal–organic complexation in on-surface synthesis
Source: Chem Sci. 2025 Jan 13;16(7):3198–210. doi: 10.1039/d4sc07550a (PMC11744327; doi:10.1039/d4sc07550a)
Supplement: SC-016-D4SC07550A-s001 [file SC-016-D4SC07550A-s001.pdf]

Supplementary Information (SI) for Chemical Science.
This journal is © The Royal Society of Chemistry 2025

# Supplementary Information

## Competing pathways to aromaticity governed by amine dehydrogenation and metal-organic complexation in on-surface synthesis

Andrés Lombana[1], Songpol Chaunchaiyakul[2], Olivier Chuzel[3,*], Denis Hagebaum-Reignier[3], Jean-Luc Parrain[3,*], Franck Bocquet[1], Laurent Nony[1], Christian Loppacher[1], Federica Bondino[4], Elena Magnano[4,5], Hiroshi Imada[2], Emiko Kazuma[2], Yousoo Kim[2], Luca Giovanelli[1,*], Sylvain Clair[1,*]

[1] *Aix Marseille University, Université de Toulon, CNRS, IM2NP, 13013 Marseille, France*
[2] *Surface and Interface Science Laboratory, RIKEN, 2-1 Hirosawa, Wako, Saitama 351-0198, Japan*
[3] *Aix Marseille Univ, CNRS, Centrale Med, ISM2, Marseille, France*
[4] *CNR - Istituto Officina dei Materiali (IOM), AREA Science Park, Basovizza, 34149 Trieste, Italy*
[5] *Nanotechnology Research Laboratory, Faculty of Engineering, University of Sydney, Camperdown 2006, Australia*

Corresponding authors: L.G. luca.giovanelli@im2np.fr; S.C. sylvain.clair@cnrs.fr

## Table of Contents

Additional STM images ........ 2
Additional XPS data ........ 7
DFT modeling of XPS data ........ 9
Precursor synthesis and characterization ........ 14
References ........ 19

## Additional STM images

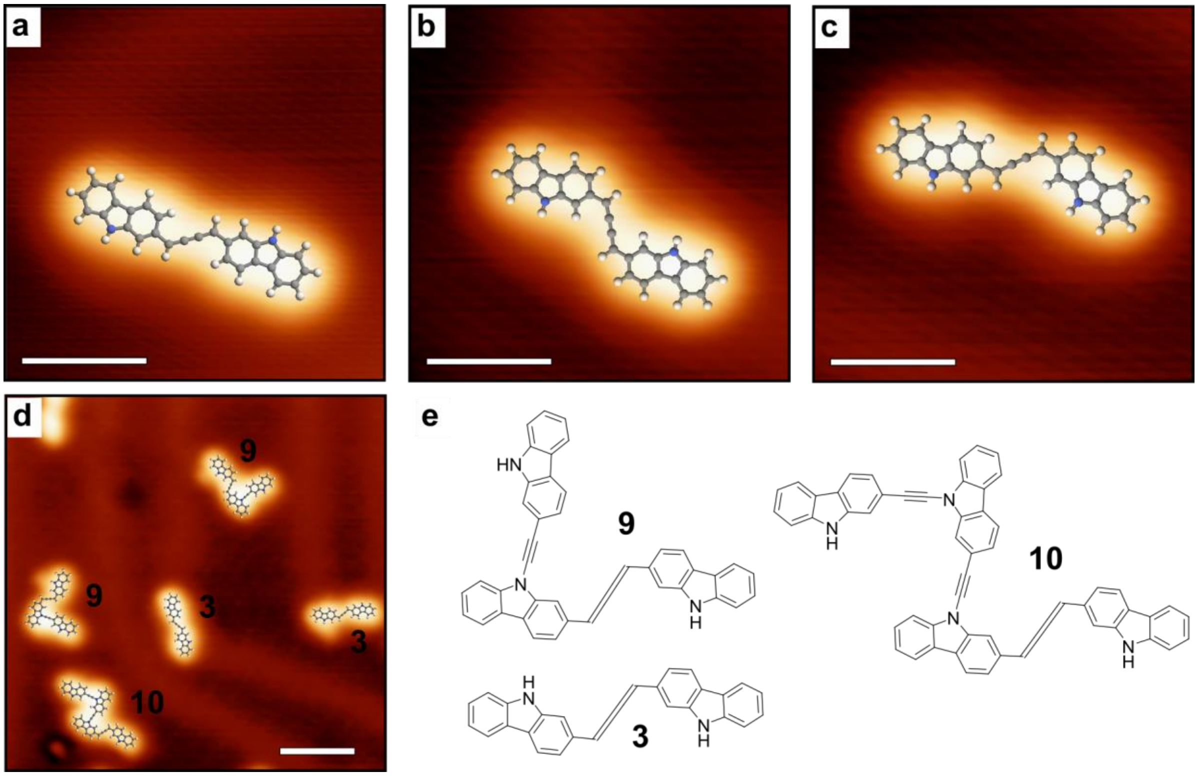


Figure S1. LT-STM images with superimposed models obtained on Au(111) after annealing at 200 °C. (a-c) Cumulene dimers with different conformations. (d) Oligomers including ynamine links and (e) corresponding proposed structures. Scale bars: (a-c) 1 nm; (d) 4 nm.

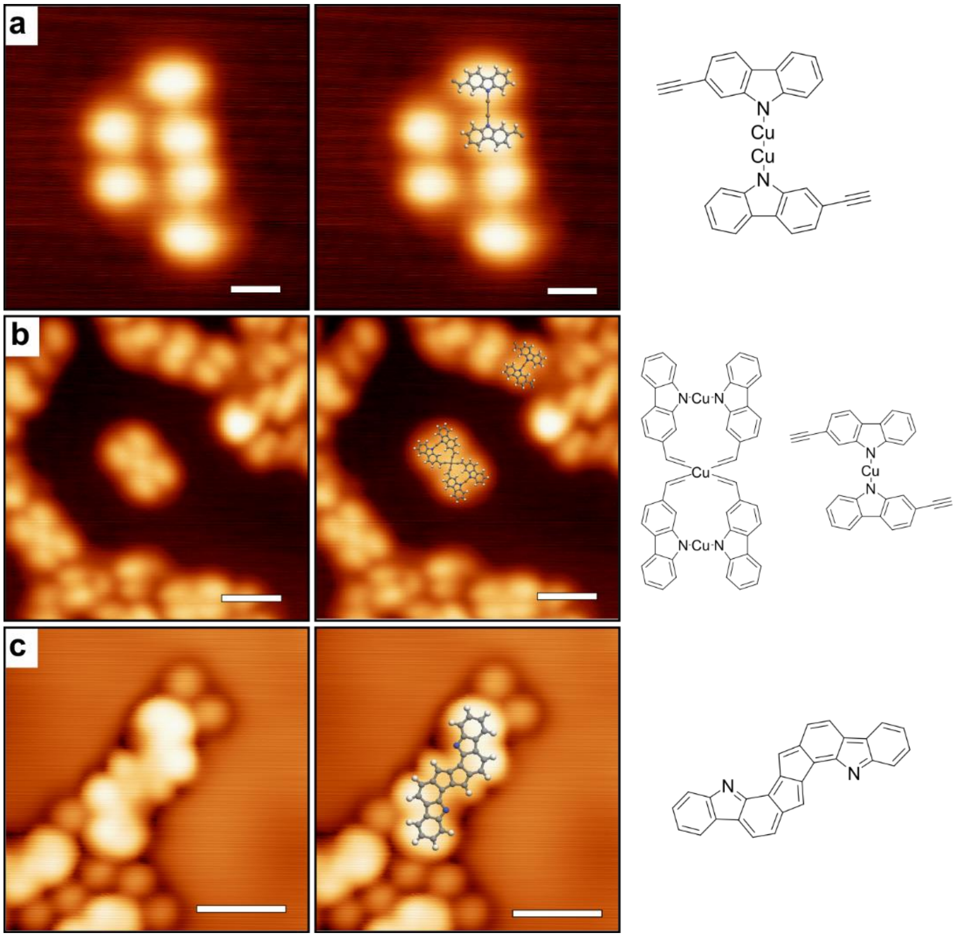


Figure S2. LT-STM images of the reaction products of **2-DCV-cbz** deposited on Cu(111), as shown in Fig. 4 of the main manuscript. The raw STM images are shown together with the corresponding molecular models and also superimposed to the images. (a) RT deposition. (b) Annealed to 200°C. (c) Hot substrate (200°C) deposition. Scale bars: (a,c) 1 nm, (b) 2 nm.

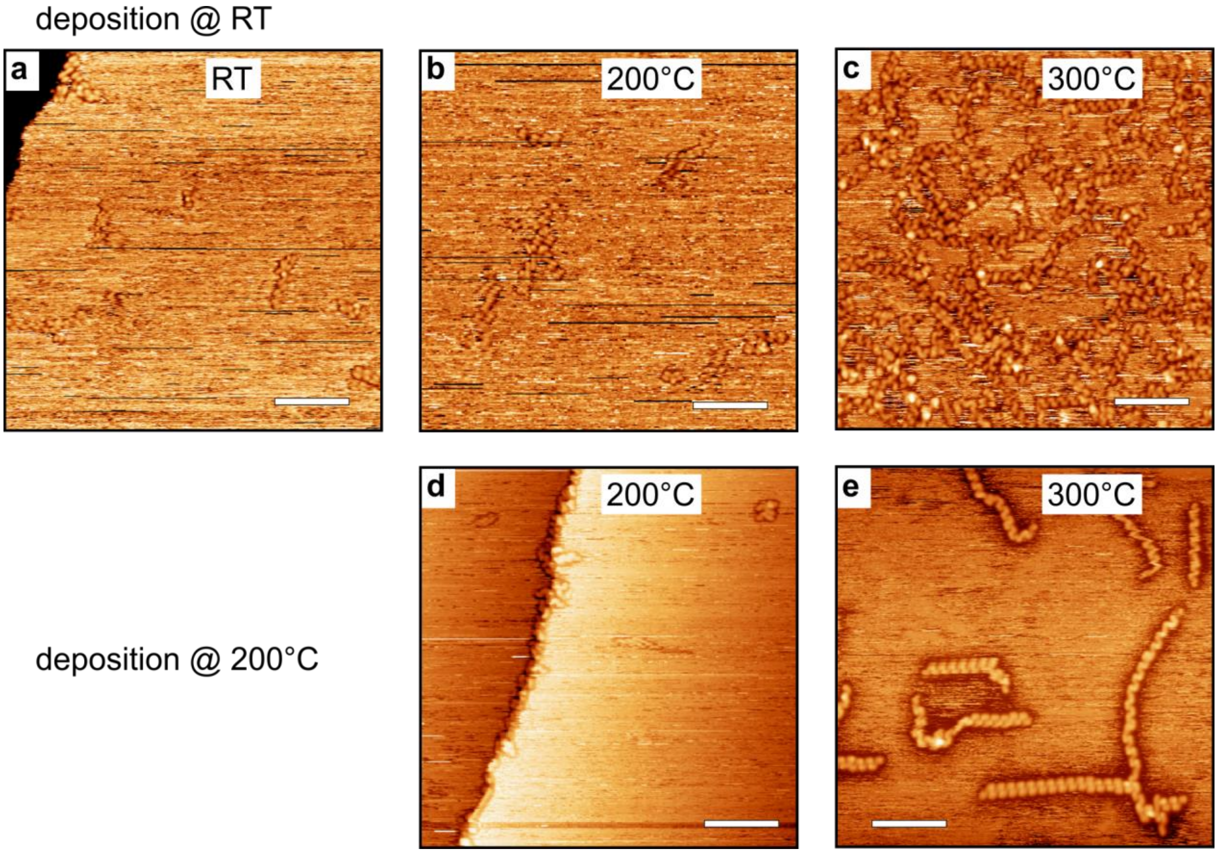


Figure S3. STM images acquired at room temperature obtained on Cu(111) after deposition at room temperature (a-c) or 200°C (d-e) and after annealing at 200°C (b,d) and 300°C (c,e). Stable structures (non-diffusing) are obtained only after annealing at 300°C. Scale bars: 10 nm.

On Au(111) after deposition and after annealing, all species are highly diffusing and not observable by STM at room temperature.

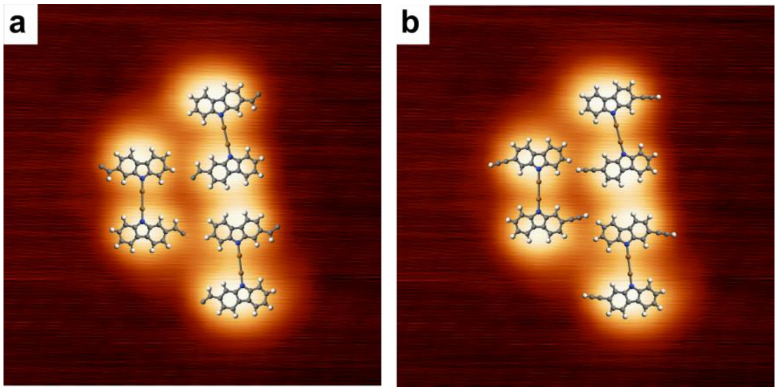


Figure S4. LT-STM images of **2-DCV-cbz** on Cu(111) obtained after RT deposition with superimposed models corresponding to the vinylidene carbene (a) or to the alkyne derivatives (b).

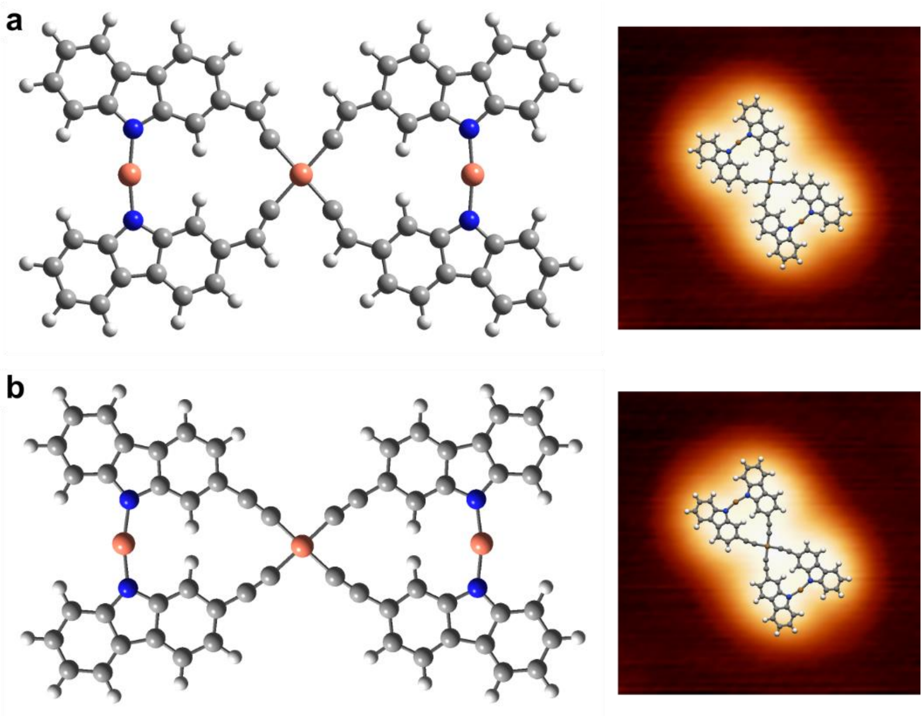


Figure S5. Tetramer structures on Cu(111) obtained after RT deposition and 200 °C annealing and corresponding STM images with superimposed models. The vinylene-based complex (a) is barely distinguishable from the alkyne-based one (b). The structures were relaxed with the semi-empirical method PM6. Because cumulene formation was not observed on Cu(111) in these conditions, the proposed structures differ from other similar cumulene-based tetramers previously observed.[1,2]

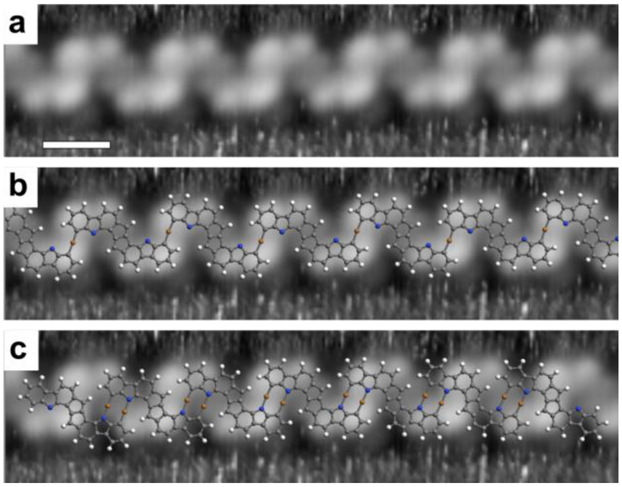


Figure S6. (a) RT-STM image of the polymer chain (scale bar: 1 nm). (b) Superimposed model of the polymer structure with C-Cu-C organometallic bridges as calculated with the semi-empirical PM6 model providing a periodicity of 1.38 nm. (c) For comparison another structure with C-Cu-N bridges is shown, providing a smaller periodicity of 1.16 nm. The measured experimental periodicity is (1.35 ± 0.1) nm.

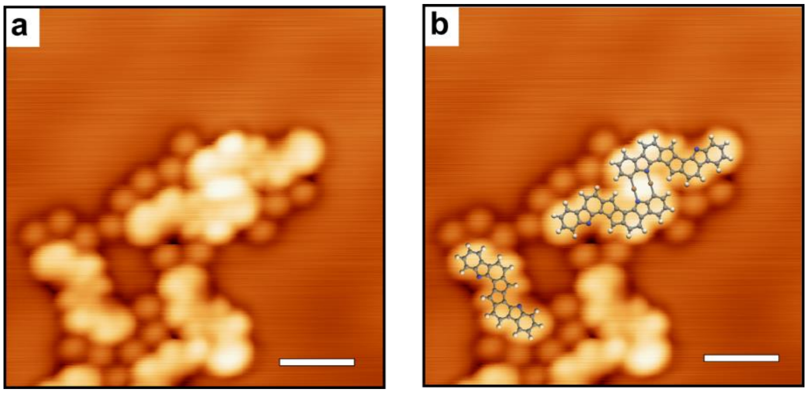


Figure S7. LT-STM image obtained on Cu(111) after deposition at 200 °C. The upper structure corresponds to two pentalene dimers linked by a double C-Cu-N bridge. Scale bars: 2 nm.

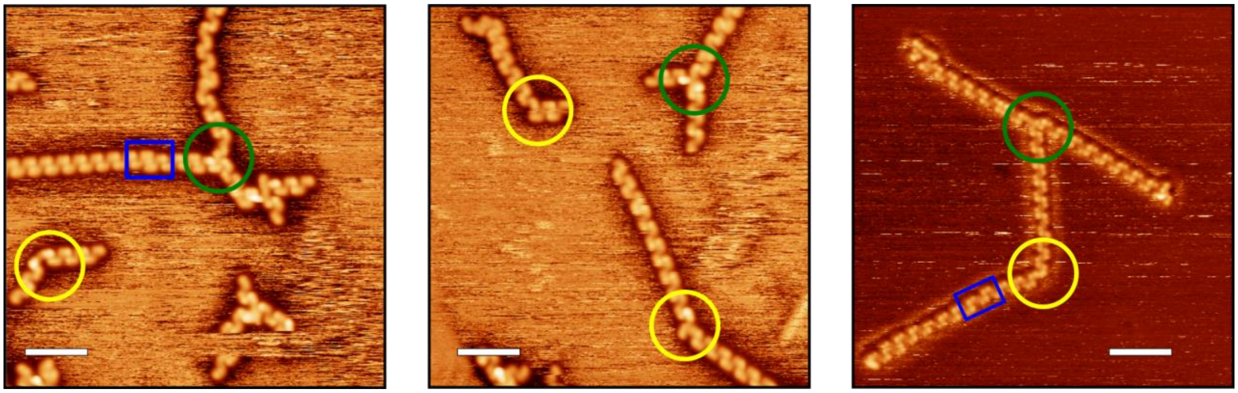

Figure S8. RT-STM images of the polymer obtained on Cu(111) after deposition at 200 °C and annealing at 300°C, showing the different kinds of defects. Blue: point defect in the periodicity. Yellow: curved section. Green: threefold interconnection. Scale bars: 5 nm.

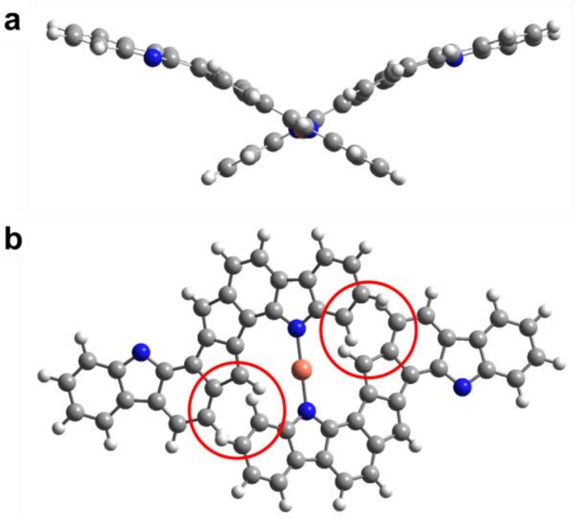


Figure S9. DFT-relaxed structure of a Cu-coordinated di-pentalene compound. Due to the steric hindrance the structure is intrinsically 3D and cannot form on a surface.[3] (a) Side view. (b) Top view.

## Additional XPS data

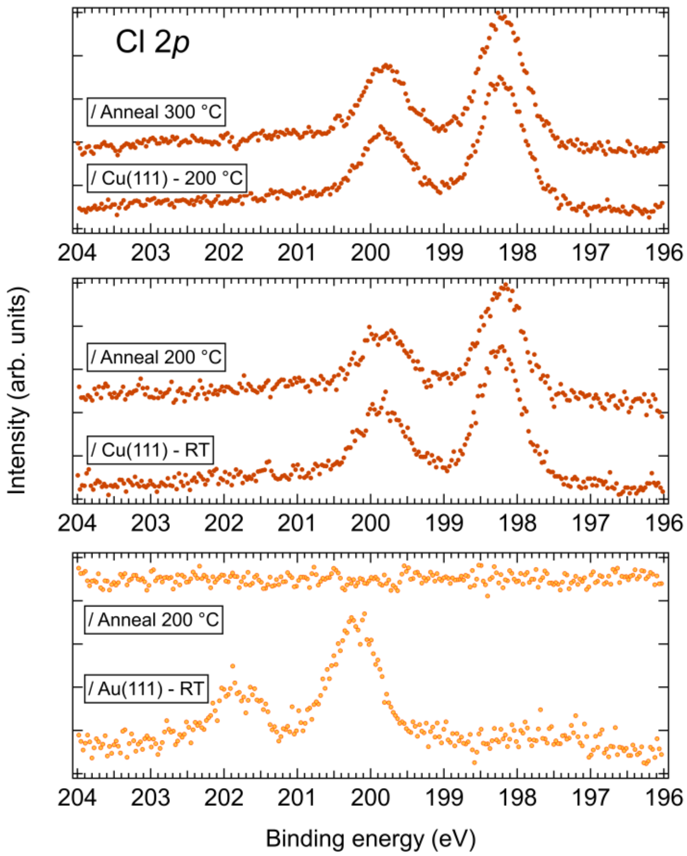


Figure S10. Cl 2p core level spectra measured for different sample preparations from the **2-DCV-cbz** precursor. On Au(111), at RT the core level position is representative of the pristine molecule. On Cu(111) all the samples present a spectrum in line with Cl atoms having left the **2-DCV-cbz** precursors and adsorbed on the metal surface.

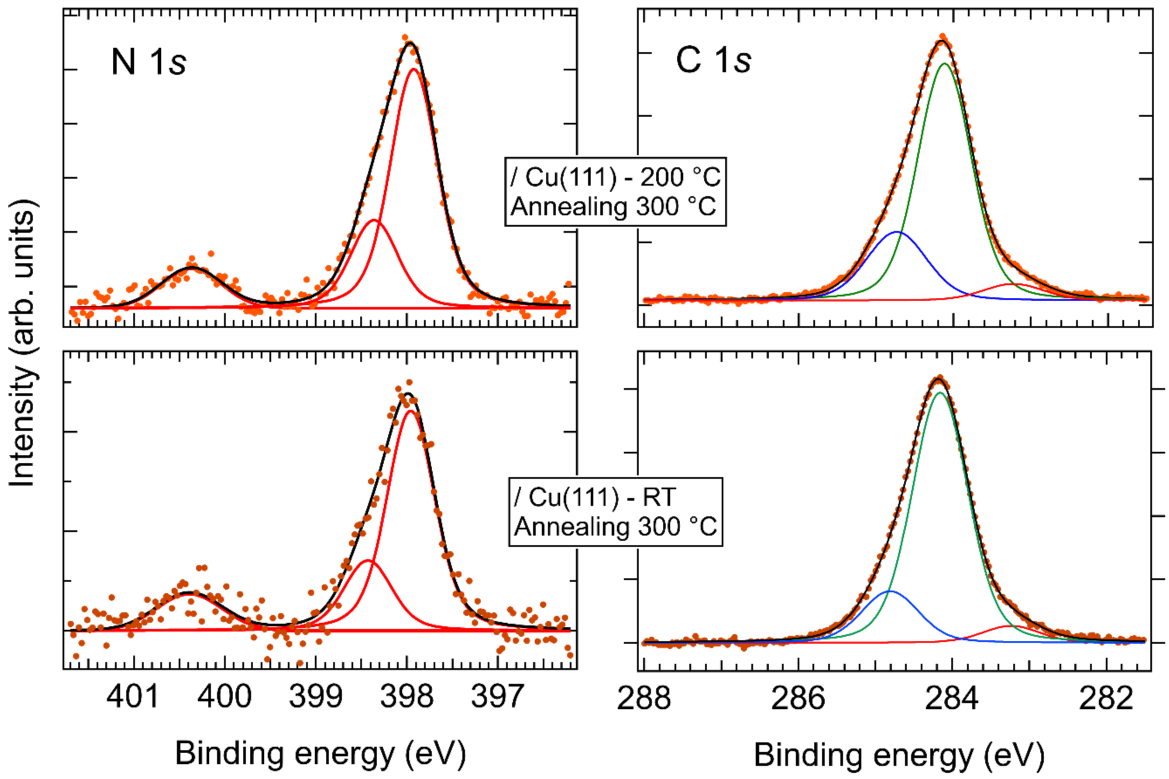


Figure S11. N 1s and C 1s spectra of **2-DCV-cbz** / Cu(111). Bottom: RT-deposition and annealing at 300 °C. Top: 200 °C-deposition and annealing at 300 °C. Both sets are barely distinguishable.

## DFT modeling of XPS data

Core-level shifts

Density functional theory have been used to study the line shape modifications of the C 1s spectra. The isolated molecules' Kohn-Sham (KS) energies were convoluted with gaussian shape curves. The resulting model spectra were compared to the experimental data to validate the structural models. The sum curves were shifted to different values to account for different molecule-substrate interactions. Gaussian widths were also adapted to account for different degrees of sample inhomogeneity coming from disorder and molecule-substrate interaction. In order to follow the origin of the line shape modifications, the C 1s calculated energies of different atoms within the molecules are color-coded according to the molecular models next to each spectrum. For instance, the pink Kohn-Sham energy in **2-DCV-cbz** / Au(111) deposited at RT corresponds to the C atoms bound to Cl. To better capture the trends, the atoms across the molecules were color-grouped according to their energies. The results shown in Fig. S12 are used to interpret the least-square fits presented in Fig. 6 (main manuscript text).

Au(111)

**RT deposition**. The C 1s KS energies of the pristine **2-DCV-cbz** can be divided in three main regions: i) the main asymmetric peak made by a number of C-C and C-H sites of the carbazole moiety (green) plus two energy-separated sites (cyan); ii) the shoulder to the main peak formed by the C-N (blue) sites; iii) the C-Cl component at highest BE (pink).

**200 °C annealing.** The C-Cl component obviously disappears; the C-N remains almost unchanged while the vinyl-related sites shift closer to the rest of the KS states.

Cu(111)

**RT deposition.** For the metal-organic complex, the C-N component is still well separated but relatively closer in energy from the rest of the KS states thus becoming an asymmetry to the main peak. The agreement with the experimental data, although still satisfying, is less good than for Au(111), probably due to the presence of different kinds of complexes. Three components are used in the least-square fit (Fig. 6b main manuscript). The red one accounting for the small asymmetry at low BE that can be assigned to the radical C atoms that were initially bonded to Cl.[4]

**300 °C annealing.** For the organometallic polymer the C-N component is not clearly shifted from the rest of the KS states but rather part of a group of states (cyan) accounting for the asymmetry to the main peak. On the other hand, two low BE states (red) are related to the C-Cu bonding.[4] Although the agreement with the experiment is again worse than for Au(111), the main characteristics of the evolution from the RT to the 300 °C annealing spectra (see also the inset) are significantly well captured by the model.

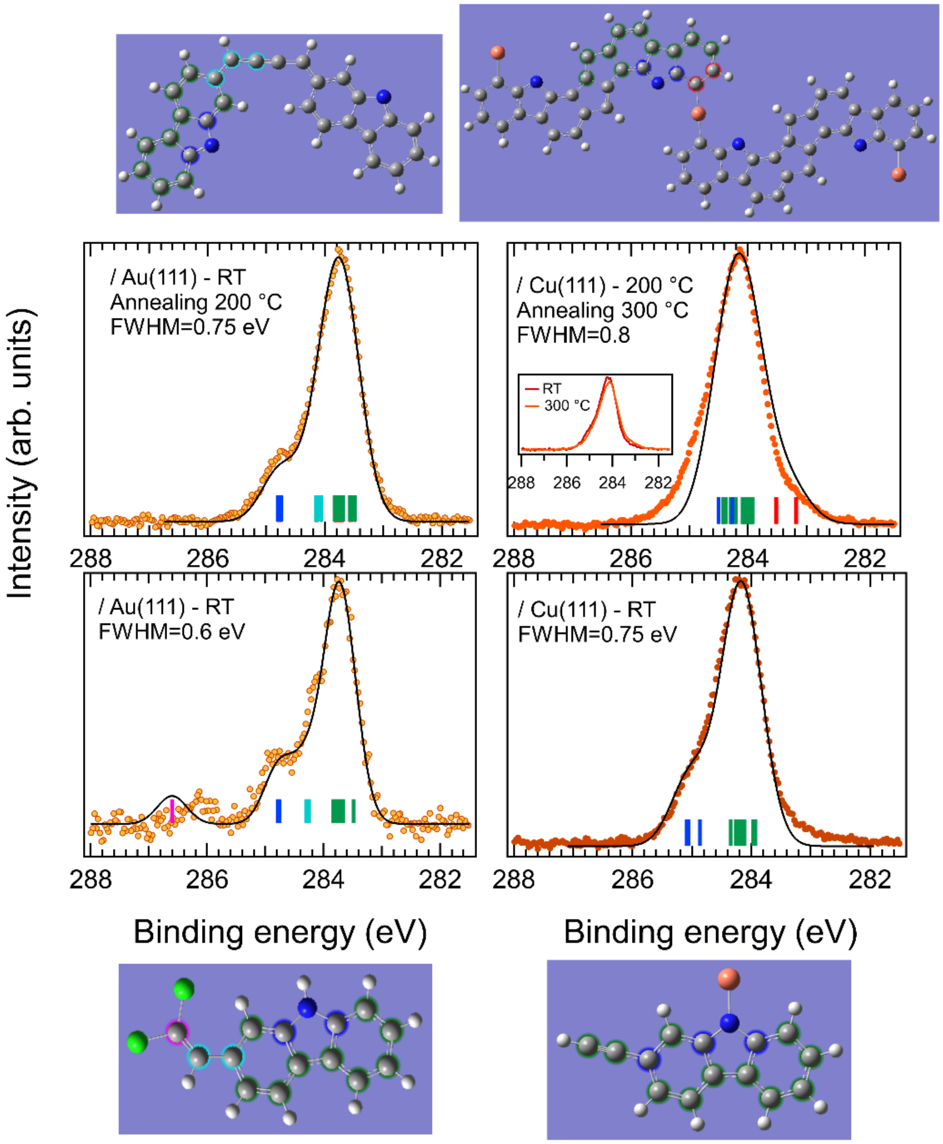


Figure S12. DFT modeling of the C 1s core levels of four selected samples of **2-DCV-cbz** deposited on (left) Au(111) and (right) Cu(111). The bottom spectra are as-taken from RT deposited samples. The top spectra are annealed samples as indicated. The superposed black lines are from convoluted gaussians (the FWHM's are indicated) centered at the C 1s orbital states calculated from the corresponding molecular models. Indication of the origin of each state within the molecular models is color-coded. For instance, for **2-DCV-cbz** deposited at RT on Au(111), the shoulder at 284.75 eV is due to blue-encircled C-N atoms. The inset in the upper-right spectrum highlights the line shape changes between RT and 300 °C-annealed samples on Cu(111).

Table S1: Least-square fit of the XPS spectra

| | N 1s | | | C 1s | | |
|---|---|---|---|---|---|---|
| Sample | BE (eV) | GW (eV) | Origin | BE (eV) | GW (eV) | Origin |
| / Au(111) - RT | 399.35 | 0.62 | N−H | 283.72 | 0.57 | C−C |
| | | | | 284.25 | 0.48 | C−C |
| | | | | 284.83 | 0.57 | C−N |
| | | | | 286.17 | 0.48 | C−Cl |
| / Au – RT + 200 °C | 399.43 | 0.78 | N−H | 283.58 | 0.58 | C−C |
| | | | | 283.99 | 0.58 | C−C |
| | | | | 284.70 | 0.58 | C−N |
| / Cu(111) - RT | 398.54 | 0.59 | N−Cu | 283.10 | 0.68 | |
| | 397.88 | 0.59 | C=N−C | 284.18 | 0.70 | C−C |
| | | | | 284.34 | 0.68 | C−N |
| / Cu – RT + 200 °C | 398.54 | 0.69 | N−Cu | 283.34 | 0.74 | |
| | 397.87 | 0.30 | C=N−C | 284.17 | 0.71 | C−C |
| | | | | 284.86 | 0.74 | C−N |
| / Cu(111) - 200 °C | 397.92 | 0.49 | C=N−C | 283.19 | 0.79 | C−Cu |
| | 398.35 | 0.49 | Shake up | 284.13 | 0.71 | C−C |
| | 400.41 | 0.80 | Shake up | 284.76 | 0.79 | C−N |
| / Cu -200 °C+300 °C | 397.92 | 0.50 | C=N−C | 283.22 | 0.81 | C−Cu |
| | 398.36 | 0.50 | Shake up | 284.12 | 0.71 | C−C |
| | 400.37 | 0.78 | Shake up | 284.74 | 0.81 | C−N |

Binding energies (BE), Gaussian widths in the Voigt line shape (GW) and assignments (on the base of the DFT analysis shown in Fig. S12). The Lorentzian contribution was 0.2 eV for all C and N spectra.

Satellite features

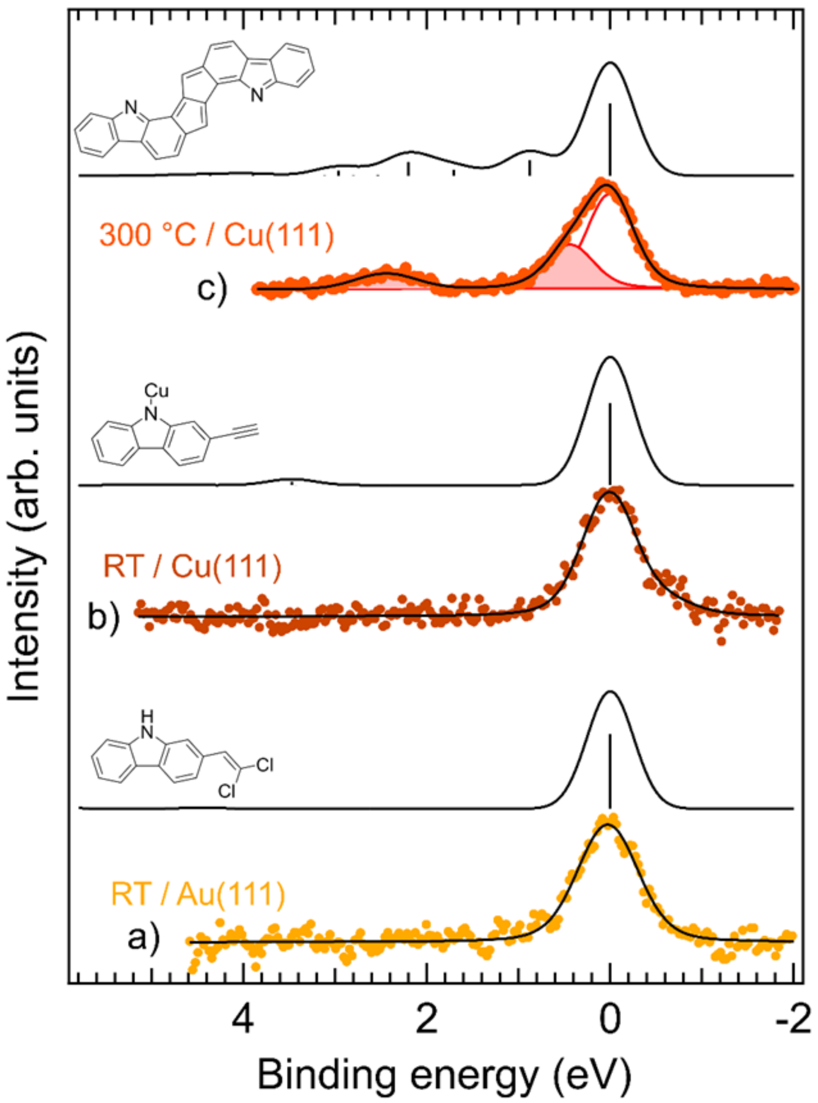


Figure S13. Modeling of the N 1s shake up. The experimental data (markers and fitting lines) are compared with ECH TD-DFT calculations of representative model compounds. For a better comparison, all spectra are aligned at the main line BE of RT / Cu(111). (a) The HOMO-LUMO gap for the **2-DCV-cbz** precursor is evaluated to 4.1 eV by TDDFT. Accordingly, satellites are vanishing for the deposition on Au(111). (b) The RT metal-organic complex on Cu(111) shows very small shake up feature, possibly undistinguishable within the background noise level. (c) The pentalene dimer displays shake up features in good agreement with the spectrum of the polymeric chains obtained at high annealing temperature.

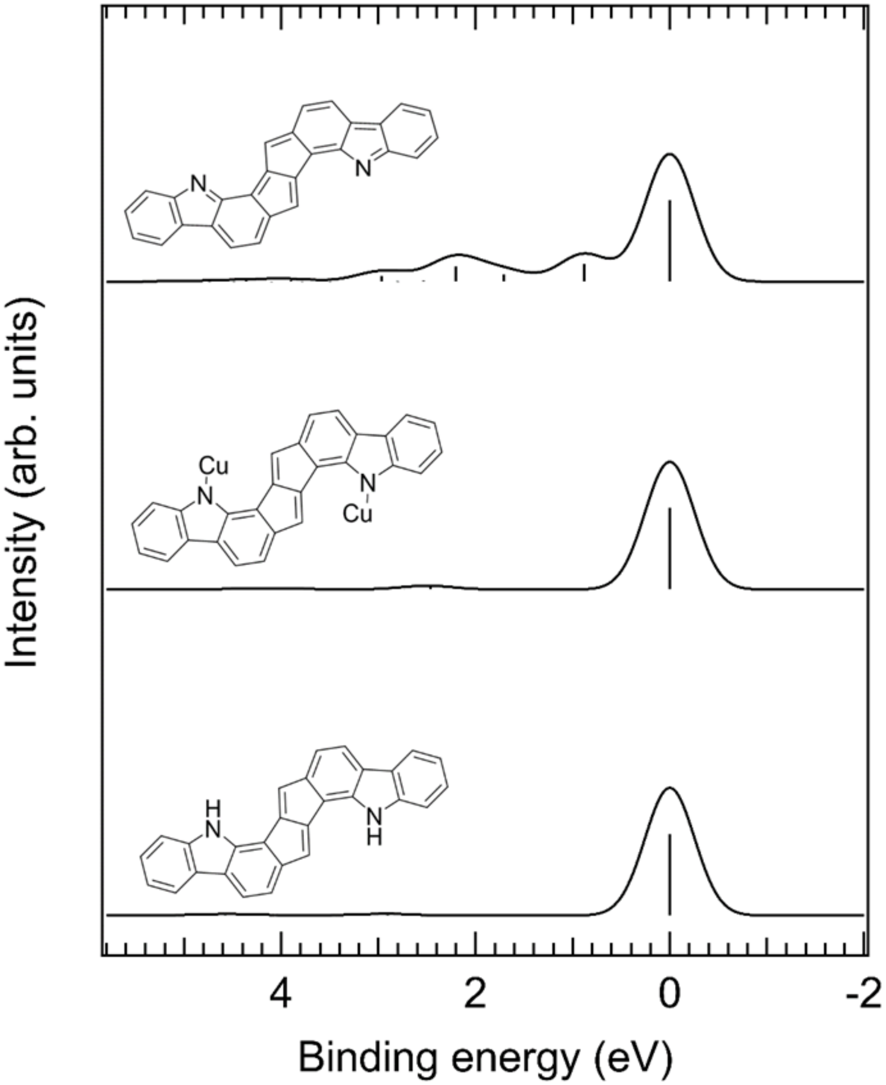


Figure S14. Modeling of the N 1s shake up for the virtual pentalene dimers hydrogenated (bottom) or with Cu complexation (middle) and comparison with the actual dehydrogenated dimer (top). The calculated small and vanishing satellite features in the hydrogenated and the Cu-coordinated dimers do not compare with the measured spectrum.

# Precursor synthesis and characterization

Synthesis

Unless otherwise noted, reactions were carried out under an argon atmosphere using syringes, cannula, oven-dried glassware and dry solvents. Dry toluene and $CH_2Cl_2$ were obtained from a MBRAUN solvent purification system MB SPS-800. All reagents and commercial anhydrous solvents were purchased from Sigma-Aldrich and used as received without further purification.

Reactions were monitored by thin layer chromatography on Merck Kieselgel 60 F254 0.2 mm plates. Visualization was accomplished using ultraviolet light (254 and/or 365 nm). Solvents were removed under reduced pressure with a rotary evaporator (BUCHI RH II). Purifications were routinely performed by column chromatography in silica gel (40-63 μm).

Characterization

NMR data were recorded at 298 ± 3 K on a BRUKER Avance III nanobay 300 MHz spectrometer equipped with a BBFO+ probe. $CDCl_3$ or DMSO were used as deuterated solvents. Chemical shifts for $^1$H and $^{13}$C NMR are reported in ppm (δ) relative to residual solvent signals. Coupling constants are given in Hertz (Hz). High resolution mass spectra (HRMS) were recorded in triplicate on a Waters Synapt G2 HRMS apparatus using electrospray (ESI) ionization source. The instrument was operated in the positive ion mode and spectra were obtained with a time-of-flight (TOF) detector.

Carbazole derivatives synthesis:

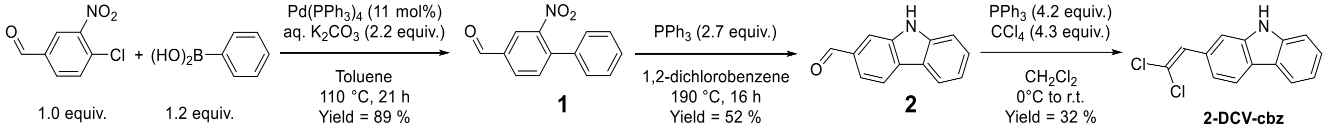


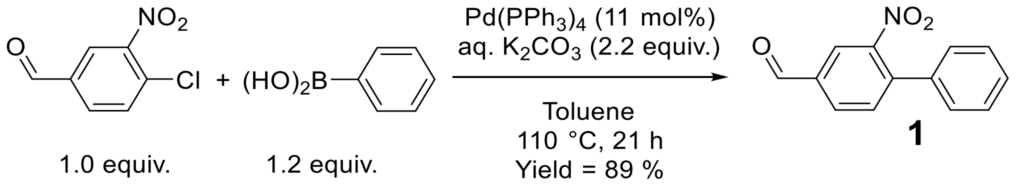


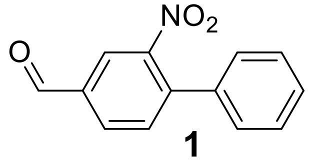


2-nitro-[1,1'-biphenyl]-4-carbaldehyde

3-chloro-4-nitrobenzaldehyde (2.035 g, 11.0 mmol, 1.0 equiv.), phenylboronic acid (1.558 g, 12.8 mmol, 1.2 equiv.) and potassium carbonate (3.259g, 23.6 mmol, 2.2 equiv.) were loaded into a Schlenk under argon. Thaw-freeze pump degassed water (9 mL) and toluene (14 mL) were transferred to the Schlenk under argon, followed by tetrakis(triphenylphosphine) palladium (139 mg, 11 mol%) and sparged with bubbling Ar for 5 min. The mixture was refluxed (T = 110 °C) for 23 h, cooled, filtered, and washed with $Et_2O$ (3 x 50 mL). The filtrate was then washed with water (2 x 50 mL), brine (20 mL), dried over sodium sulfate, then filtrated. Upon concentration *in vacuo*, the pure product readily crystallized (2.208 g, 89 % yield) as a yellow solid.

$^{1}$H NMR (400 MHz, δ, Chloroform-*d*) 10.10 (s, 1H), 8.33 (d, $^{4}J$ = 1.6 Hz, 1H), 8.12 (dd, $^{3}J$ = 7.9 Hz, $^{4}J$ = 1.7 Hz, 1H), 7.65 (d, $^{3}J$ = 7.9 Hz, 1H), 7.54-7.39 (m, 3H), 7.37-7.33 (m, 2H).

Spectroscopic data and analytical characterization are consistent with the literature.[5]

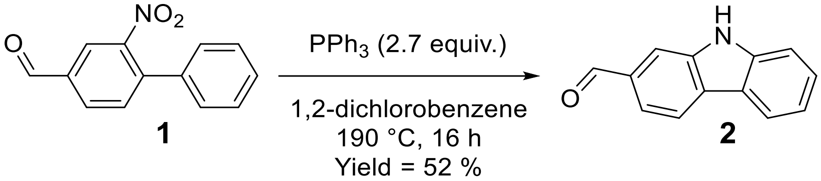


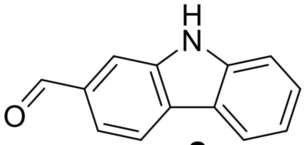

**2**

9*H*-carbazole-2-carbaldehyde

2-nitro-[1,1'-biphenyl]-4-carbaldehyde **1** (69 mg, 0.30 mmol) and triphenylphospine (210 mg, 0.81 mmol) were dissolved in thaw-freeze pump degassed 1,2-dichlorobenzene (3 mL). The mixture was stirred at reflux for 16 h, then concentrated *in vacuo*. Purification by flash chromatography on silica gel ($CH_2Cl_2$:EtOAc; 9:1) gave the pure product as dark yellow crystals (31 mg, 52 % yield).

$^{1}$H NMR (400 MHz, Chloroform-*d*) δ 10.14 (s, 1H), 8.36 (bs, 1H), 8.20 (d, $^{3}J$ = 8.0 Hz, 1H), 8.14 (d, $^{3}J$ = 7.9 Hz, 1H), 7.99 (s, 1H), 7.77 (dd, $^{3}J$ = 8.0 Hz, $^{4}J$ = 1.3 Hz, 1H), 7.55 – 7.47 (m, 2H), 7.30 (ddd, $^{3}J$ = 8.0 Hz, 6.3 Hz, $^{4}J$ = 1.8 Hz, 1H).

Spectroscopic data and analytical characterization are consistent with the literature.[6]

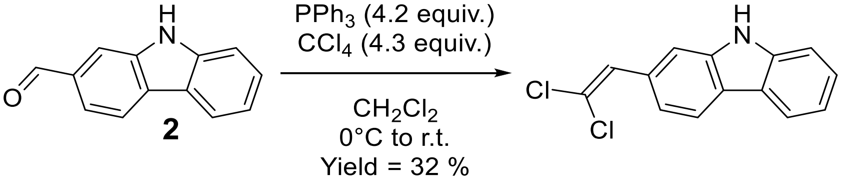


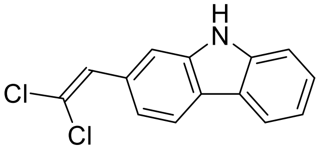

2-(2,2-dichlorovinyl)-9*H*-carbazole

**2-DCV-cbz**

Procedure adapted from the literature.[7]

9*H*-carbazole-2-carbaldehyde **2** (47 mg, 0.24 mmol, 1.0 equiv.) and triphenylphosphine (266 mg, 1.01 mmol, 4.2 equiv.) are dissolved in dichloromethane (5 mL) under argon and stirred at 0 °C in an ice-water bath. Tetrachloromethane (0.1 mL, 1.03 mmol, 4.3 equiv.) is then added dropwise, and the reaction allowed to warm to room temperature and followed by thin layer chromatography. Upon conversion of the starting material, the solution is poured into water, extracted with dichloromethane 3 times and the combined organic phases are dried over $Na_2SO_4$ and evaporated. Purification is performed by flash chromatography on silica gel ($CH_2Cl_2$: petroleum ether; 2:8 to 1:0) and yielded to **2-DCV-cbz** (20 mg, 0.08 mmol, 33 % yield) as colorless needles.

$^{1}$H NMR (300 MHz, DMSO-$d_6$) δ 11.36 (s, 1H), 8.13 (d, $^{3}J$ = 8.2 Hz, 1H), 8.12 (d, $^{3}J$ = 7.9 Hz, 1H), 7.80 (s, 1H), 7.49 (d, $^{3}J$ = 8.2 Hz, 1H), 7.45 – 7.36 (m, 3H), 7.17 (td, $^{3}J$ = 8.0 Hz, $^{4}J$ = 1.1 Hz, 1H).

$^{13}$C NMR (75 MHz, DMSO-$d_6$) δ 140.5, 139.4, 130.1, 129.8, 126.1, 122.7, 121.9, 120.4, 120.1, 119.5, 118.8, 117.5,111.0, 110.9.

HRMS (Negative mode, TOF ESI$^-$, MeOH): *m/z* calcd for $C_{14}H_8NCl_2^-$ = 260.0039; found 260.0042.

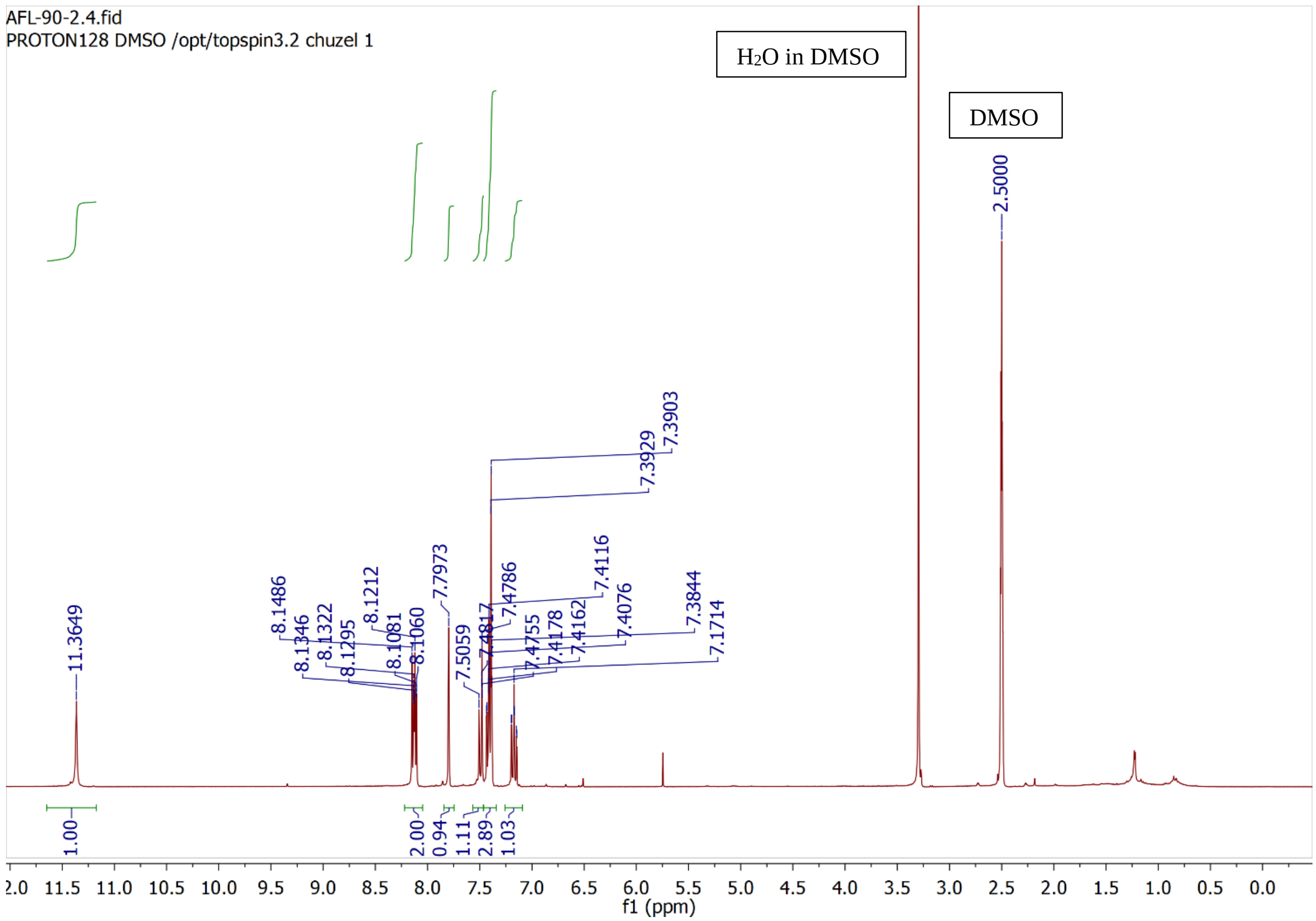

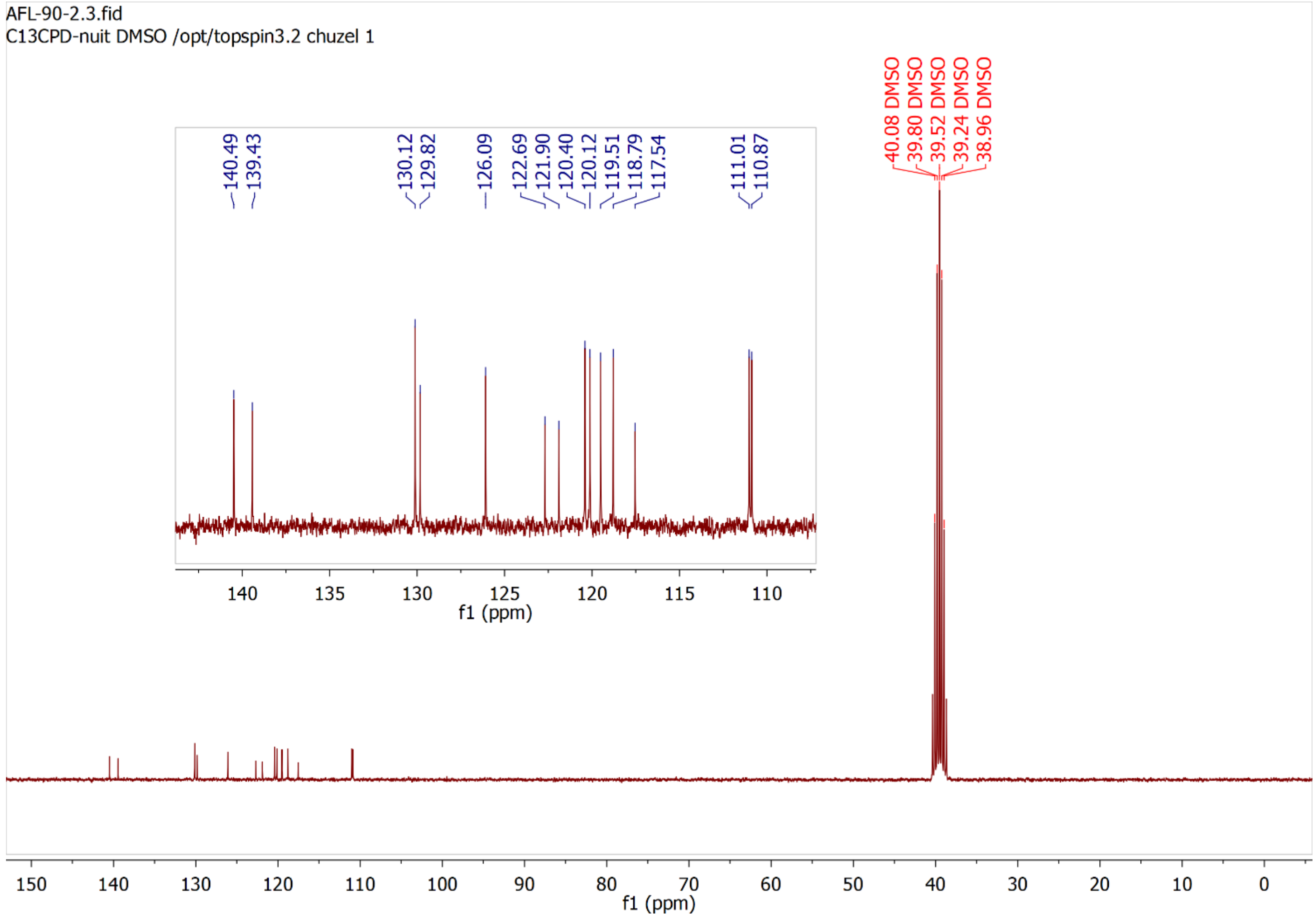


**XRD Structure of 2-DCV-cbz**

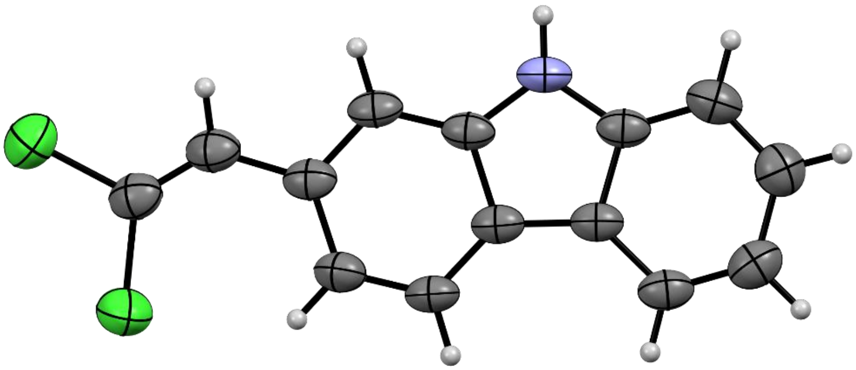

Table S2. Crystal data and structure refinement for **2-DCV-cbz**

| | **2-DCV-cbz** |
|---|---|
| Identification code | CCDC 2303753 |
| Empirical formula | $C_{14}H_9Cl_2N$ |
| Formula weight | 262.12 |

| | |
|---|---|
| Temperature/K | 293 |
| Crystal system | Orthorhombic |
| Space group | $Pna2_1$ |
| a/Å | 7.9976(2) |
| b/Å | 5.7344(10) |
| c/Å | 25.2101(7) |
| α/° | 90 |
| β/° | 90 |
| γ/° | 90 |
| Volume/$Å^3$ | 1156.17(5) |
| Z | 4 |
| $\rho_{calc}g/cm^3$ | 1.506 |
| $\mu/mm^{-1}$ | 4.815 |
| F(000) | 536.0 |
| Crystal size/$mm^3$ | 0.32 × 0.08 × 0.015 |
| Radiation | CuKα (λ = 1.54184) |
| 2Θ range for data collection/° | 7.012 to 141.986 |
| Index ranges | -7 ≤ h ≤ 9, -4 ≤ k ≤ 6, -21 ≤ l ≤ 30 |
| Reflections collected | 3369 |
| Independent reflections | 1754 [$R_{int}$ = 0.0264, $R_{sigma}$ = 0.0302] |
| Data/restraints/parameters | 1754/1/154 |
| Goodness-of-fit on $F^2$ | 1.063 |
| Final R indexes [I>=2σ (I)] | $R_1$ = 0.0395, $wR_2$ = 0.1042 |
| Final R indexes [all data] | $R_1$ = 0.0407, $wR_2$ = 0.1057 |
| Largest diff. peak/hole / e $Å^{-3}$ | 0.34/-0.30 |
| Flack parameter | 0.026(19) |

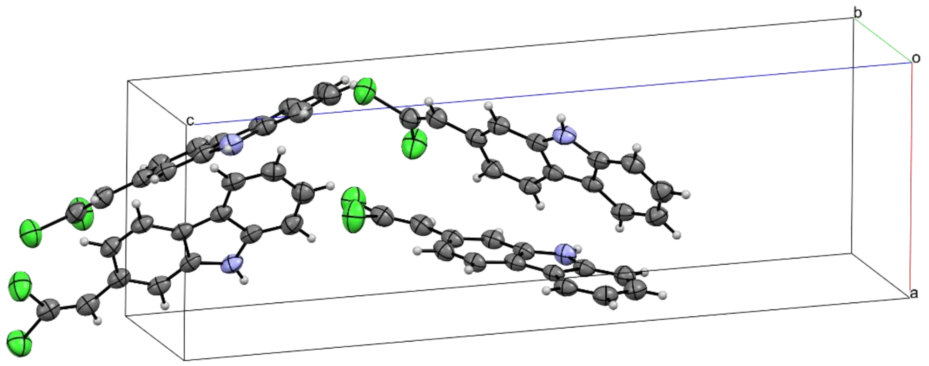


Figure S15. XRD packing structure of **2-DCV-cbz**.

From the presented structure it is evident that no H-bond acceptor is present on the vicinity of the only H-bond donor (N–**H**) and the shortest distance between a Cl and a H atom is of 3.073 Å.

## References


(1) Zhang, C.; Sun, Q.; Kong, H. H.; Yuan, C. X.; Xu, W. On-surface stereoconvergent synthesis, dimerization and hybridization of organocopper complexes. *Science China-Chemistry* **2019,** *62*, 126-132.

(2) Cirera, B.; Riss, A.; Mutombo, P.; Urgel, J. I.; Santos, J.; Di Giovannantonio, M.; Widmer, R.; Stolz, S.; Sun, Q.; Bommert, M.; et al. On-surface synthesis of organocopper metallacycles through activation of inner diacetylene moieties. *Chem. Sci.* **2021,** *12*, 12806-12811.

(3) Kinikar, A.; Wang, X. Y.; Di Giovannantonio, M.; Urgel, J. I.; Liu, P. C.; Eimre, K.; Pignedoli, C. A.; Stolz, S.; Bommert, M.; Mishra, S.; et al. Sterically Selective 3+3 Cycloaromatization in the On-Surface Synthesis of Nanographenes. *Acs Nanoscience Au* **2023,** *4*, 128-135.

(4) Simonov, K. A.; Vinogradov, N. A.; Vinogradov, A. S.; Generalov, A. V.; Zagrebina, E. M.; Martensson, N.; Cafolla, A. A.; Carpy, T.; Cunniffe, J. P.; Preobrajenski, A. B. Effect of Substrate Chemistry on the Bottom-Up Fabrication of Graphene Nanoribbons: Combined Core-Level Spectroscopy and STM Study. *J. Phys. Chem. C* **2014,** *118*, 12532-12540.

(5) Kim, Y. M.; Yu, S. Palladium(0)-catalyzed amination, Stille coupling, and Suzuki coupling of electron-deficient aryl fluorides. *J. Am. Chem. Soc.* **2003,** *125*, 1696-1697.

(6) Freeman, A. W.; Urvoy, M.; Criswell, M. E. Triphenylphosphine-mediated reductive cyclization of 2-nitrobiphenyls: A practical and convenient synthesis of carbazoles. *J. Org. Chem.* **2005,** *70*, 5014-5019.

(7) Tu, Y. L.; Zeng, X. Z.; Wang, H.; Zhao, J. F. A Robust One-Step Approach to Ynamides. *Org. Lett.* **2018,** *20*, 280-283.
